# Novel molecular subgroups for clinical classification and outcome prediction in childhood medulloblastoma: a cohort study

**DOI:** 10.1016/S1470-2045(17)30243-7

**Published:** 2017-07

**Authors:** Edward C Schwalbe, Janet C Lindsey, Sirintra Nakjang, Stephen Crosier, Amanda J Smith, Debbie Hicks, Gholamreza Rafiee, Rebecca M Hill, Alice Iliasova, Thomas Stone, Barry Pizer, Antony Michalski, Abhijit Joshi, Stephen B Wharton, Thomas S Jacques, Simon Bailey, Daniel Williamson, Steven C Clifford

**Affiliations:** a Wolfson Childhood Cancer Research Centre, Northern Institute for Cancer Research, Newcastle University, Newcastle upon Tyne, UK; b Department of Applied Sciences, Northumbria University, Newcastle upon Tyne, UK; c Department of Haematology and Oncology Department, Great Ormond Street Hospital for Children NHS Foundation Trust, London, UK; d Neural Development Unit, UCL Institute of Child Health, London, UK; e Institute of Translational Research, University of Liverpool, Liverpool, UK; f Department of Neuropathology, Royal Victoria Infirmary, Newcastle University Teaching Hospitals NHS Foundation Trust, Newcastle upon Tyne, UK; g Sheffield Institute for Translational Neuroscience, University of Sheffield, Sheffield, UK

## Abstract

**Background:**

International consensus recognises four medulloblastoma molecular subgroups: WNT (MB_WNT_), SHH (MB_SHH_), group 3 (MB_Grp3_), and group 4 (MB_Grp4_), each defined by their characteristic genome-wide transcriptomic and DNA methylomic profiles. These subgroups have distinct clinicopathological and molecular features, and underpin current disease subclassification and initial subgroup-directed therapies that are underway in clinical trials. However, substantial biological heterogeneity and differences in survival are apparent within each subgroup, which remain to be resolved. We aimed to investigate whether additional molecular subgroups exist within childhood medulloblastoma and whether these could be used to improve disease subclassification and prognosis predictions.

**Methods:**

In this retrospective cohort study, we assessed 428 primary medulloblastoma samples collected from UK Children's Cancer and Leukaemia Group (CCLG) treatment centres (UK), collaborating European institutions, and the UKCCSG-SIOP-PNET3 European clinical trial. An independent validation cohort (n=276) of archival tumour samples was also analysed. We analysed samples from patients with childhood medulloblastoma who were aged 0–16 years at diagnosis, and had central review of pathology and comprehensive clinical data. We did comprehensive molecular profiling, including DNA methylation microarray analysis, and did unsupervised class discovery of test and validation cohorts to identify consensus primary molecular subgroups and characterise their clinical and biological significance. We modelled survival of patients aged 3–16 years in patients (n=215) who had craniospinal irradiation and had been treated with a curative intent.

**Findings:**

Seven robust and reproducible primary molecular subgroups of childhood medulloblastoma were identified. MB_WNT_ remained unchanged and each remaining consensus subgroup was split in two. MB_SHH_ was split into age-dependent subgroups corresponding to infant (<4·3 years; MB_SHH-Infant_; n=65) and childhood patients (≥4·3 years; MB_SHH-Child_; n=38). MB_Grp3_ and MB_Grp4_ were each split into high-risk (MB_Grp3-HR_ [n=65] and MB_Grp4-HR_ [n=85]) and low-risk (MB_Grp3-LR_ [n=50] and MB_Grp4-LR_ [n=73]) subgroups. These biological subgroups were validated in the independent cohort. We identified features of the seven subgroups that were predictive of outcome. Cross-validated subgroup-dependent survival models, incorporating these novel subgroups along with secondary clinicopathological and molecular features and established disease risk-factors, outperformed existing disease risk-stratification schemes. These subgroup-dependent models stratified patients into four clinical risk groups for 5-year progression-free survival: favourable risk (54 [25%] of 215 patients; 91% survival [95% CI 82–100]); standard risk (50 [23%] patients; 81% survival [70–94]); high-risk (82 [38%] patients; 42% survival [31–56]); and very high-risk (29 [13%] patients; 28% survival [14–56]).

**Interpretation:**

The discovery of seven novel, clinically significant subgroups improves disease risk-stratification and could inform treatment decisions. These data provide a new foundation for future research and clinical investigations.

**Funding:**

Cancer Research UK, The Tom Grahame Trust, Star for Harris, Action Medical Research, SPARKS, The JGW Patterson Foundation, The INSTINCT network (co-funded by The Brain Tumour Charity, Great Ormond Street Children's Charity, and Children with Cancer UK).

## Introduction

The discovery of molecular disease subgroups represents the most fundamental advance in our understanding of medulloblastoma, the most common malignant brain tumour of childhood. Current international consensus recognises four subgroups of medulloblastoma: WNT (MB_WNT_), SHH (MB_SHH_), group 3 (MB_Grp3_) and group 4 (MB_Grp4_).^1^ Each subgroup is defined empirically by genome-wide transcriptomic^2^, ^3^, ^4^, ^5^, ^6^ and DNA methylation patterns^7^, ^8^ and characterised by distinct clinicopathological and molecular features.^9^, ^10^, ^11^, ^12^ MB_WNT_ and MB_SHH_ are synonymous with WNT and SHH activating mutations.^12^ By contrast, MB_Grp3_ and MB_Grp4_ have few mutations, but have multiple DNA copy number alterations.^9^, ^10^, ^11^, ^12^

**Research in context**

**Evidence before this study**

The international consensus definition of medulloblastoma, published in 2012, recognises four primary molecular subgroups with distinct clinicopathological features: WNT (MB_WNT_), SHH (MB_SHH_), group 3 (MB_Grp3_), and group 4 (MB_Grp4_). Several studies established characteristic genome-wide transcriptomic and DNA methylomic profiles, using unsupervised class discovery techniques, which defined the consensus subgroups. These subgroups, described in the 2016 WHO classification of brain tumours, underpin current disease subclassification, research studies, and clinical trials. Profiling and class discovery studies published to date in medulloblastoma are based on cohorts typically with sample sizes less than 200 patients and, even within the consensus subgroups, significant heterogeneity of clinical and molecular features remains and many relationships to disease outcome are unresolved. Evidence from the component studies and reviews undertaken in the international consensus definition and the 2016 WHO classification, alongside our own reviews of the current literature, formed the foundation for the present study; no systematic reviews were carried out.

**Added value of this study**

We defined and characterised seven robust, reproducible, clinically significant, primary molecular subgroups within childhood medulloblastoma (in children aged up to 16 years at diagnosis), each with distinct clinicomolecular features. We propose a cross-validated, subgroup-dependent survival model that incorporates these novel subgroups, alongside established disease features and risk-factors and outperforms the disease risk-stratification schemes in current clinical use. Redistribution of disease risk using this scheme identifies substantial proportions of favourable-risk non-infant patients (>90% 5-year survival in 11% of patients) outside the MB_WNT_ subgroup (equivalent to approximately 70 patients per year in the European Union [EU]) who would be suitable for consideration of reduced intensity of therapy, and very-high-risk non-infant patients (<40% survival, 13% of patients, about 80 EU patients per year) for whom new treatment strategies should be prioritised.

**Implications of all the available evidence**

These data provide a step-change in our understanding and characterisation of molecular subgroups within medulloblastoma, with potential application to future disease subclassification, risk-stratification, and subgroup-dependent translational research.

Subgrouping is integral to the 2016 WHO medulloblastoma classification,^13^ and is used to direct treatment strategies aimed at improving cure rates (5-year survival across all four subgroups is 65–70%), and reducing long-term intellectual and neuroendocrine impairments associated with existing multimodality therapies. Patients with childhood MB_WNT_ consistently show a favourable prognosis (>90% survival^14^, ^15^) and reduced intensity risk-adapted therapies are being studied in these patients in international clinical trials,^16^ whereas SHH pathway inhibitors show promise in MB_SHH_ disease in early-phase trials,^17^ although treatment of infants (younger than 3 years at diagnosis) and young children with these inhibitors should be approached with caution, because of the risk of premature fusion of growth-plates.^18^

Substantial biological heterogeneity is evident within each non-MB_WNT_ subgroup; for instance, *TP53* mutations are associated with a poor outcome in MB_SHH_.^13^, ^19^ High-risk clinical factors (metastatic disease [M+]; large-cell, anaplastic [LCA] pathology; incomplete surgical resection [R+]; and *MYC*/*MYCN* amplification), which are currently used to stratify risk in medulloblastoma in children aged 3 years or older, were derived from cohort-wide investigations before discovery of the consensus subgroups, and thus did not consider their effect.^15^, ^16^, ^20^, ^21^

Studies that defined the four-subgroup consensus used modestly sized cohorts (typically fewer than 200 patients).^2^, ^3^, ^4^, ^5^, ^6^ In this Article, we describe comprehensive molecular profiling of clinically annotated discovery and validation cohorts totalling more than 700 tumours. We report the discovery and characterisation of seven stable and reproducible primary subgroups of childhood medulloblastoma (in patients younger than 16 years at diagnosis), which subdivide each of the classic consensus non-MB_WNT_ subgroups (MB_SHH_, MB_Grp3,_ and MB_Grp4_) into two clinically significant groups with distinct clinicomolecular features and survival outcomes.

## Methods

### Study design and participants

In this retrospective cohort study, we assessed 428 centrally reviewed, clinically annotated primary medulloblastomas from patients aged 0–16 years at diagnosis, collected from UK Children's Cancer and Leukaemia Group (CCLG) treatment centres (UK; 366 [86%]), collaborating European institutions in Budapest (Hungary; 20 [5%]) and Warsaw (Poland; 15 [4%]), and samples from the European UKCCSG-SIOP-PNET3 clinical trial (27 [6%]). As is typical for medulloblastoma, we regarded patients younger than 3 years at diagnosis as infants. 108 (26%) of 408 patient samples used were collected in 2010–14, 192 (47%) in 2000–10, 85 (21%) in 1990–2000, and the remaining 23 (6%) were collected before 1990 (18 were from the 1980s, four from the 1970s, and one was from 1968). Year of diagnosis was unavailable for 20 samples.

Tumour samples were provided by the UK CCLG as part of CCLG-approved biological study BS-2007–04; informed, written consent was obtained from parents of all patients because all assessed patients were younger than 16 years. Tumour investigations were done with approval from Newcastle North Tyneside Research Ethics Committee (study reference 07/Q0905/71); all tumour material was collected in accordance with this approval. We used 276 medulloblastomas (GSE54880) from a published tumour archive,^8^ comprising patients aged from 0–18 years at diagnosis, as an independent validation cohort. 18 post-mortem cerebellar samples were collected from the Newcastle Brain Tissue Resource and used as controls in some analyses; all samples were collected with written, informed consent.

### Procedures

We tested medulloblastoma samples with the Illumina HumanMethylation450K DNA methylation array (Illumina, San Diego, CA, USA). The Gene Expression Omnibus accession number for 450K DNA methylation array profiles we used for the determination of human medulloblastoma molecular subgroup status is GSE93646.

To identify methylation-dependent subgroups, we did unsupervised class discovery by NMF-metagene and k-means clustering, testing all combinations of 3–10 metagenes and clusters for reproducibility using bootstrapped resampling methods (250 iterations) as described previously.^7^ This analysis identified metagenes (a single score that reflects the methylation status of several CpG loci) representing the main biological effects present in the genome-wide dataset. We assessed cluster stability using the cophenetic index, a shorthand measure of the robustness of sample clustering as determined by consensus non-negative matrix factorisation (appendix p 3). We visualised clusters with t-SNE.^22^ We assigned samples classified with less than 80% confidence (by resampling procedures) as non-classifiable (NC; appendix pp 2–3).

We projected metagenes derived from our discovery cohort onto the validation cohort. Additionally, we combined the discovery and validation cohorts to do equivalent consensus clustering.

We assessed established medulloblastoma clinical, pathological, and molecular features as described previously.^7^ Briefly, we defined histopathological variants according to the WHO 2016 guidelines.^13^ We assigned metastatic status (M+) based on Chang's criteria (appendix p 3). Tumours were designated as R+ if their residuum after surgical excision exceeded 1·5 cm^2^. Pathology was centrally reviewed by three experienced neuropathologists for 380 (89%) of 428 samples, and clinical data were collated from contributing centres and reviewed centrally (appendix p 3). We assessed *MYC* and *MYCN* status by fluorescence in situ hybridisation or copy-number estimates from methylation array. We assessed *TP53, CTNNB1*, and *TERT* mutation status by Sanger sequencing. We identified subgroup-specific differentially methylated CpG loci or methylated regions (DMRs) using limma or DMRcate^23^, ^24^ (appendix p 3). RNA-seq expression data were generated for discovery cohort samples for which mRNA of sufficient quantity and quality was available. We identified subgroup-specific differentially expressed genes using DESeq2,^25^ and these genes were included in ontology enrichment analyses (appendix p 4). We identified *GFI1* mutations from RNA-seq data (appendix p 4).

MB_SHH_ mutation data were obtained from a previous study.^26^ Although 450K methylation data for MB_SHH_ subgroup assignment were not available for this sample cohort, the tightly defined age cutoff that we defined between the molecularly determined MB_SHH-Infant_ and MB_SHH-Child_ subgroups enabled us to infer subgroups for this sequencing cohort (appendix p 4).^26^ We tested recurrent MB_SHH_ mutations (*TP53, SUFU, PTCH1, SMO*, and *TERT*) and gene amplifications (*MYCN* and *GLI2*) identified by whole genome sequencing, for association with the age-defined MB_SHH-Child_ or MB_SHH-Infant_ subgroups using Fisher's exact test (appendix p 4).

### Statistical analysis

We did survival analyses (overall survival and progression-free survival) on samples from patients aged 3–16 years within our discovery cohort, who received maximal surgical resection and craniospinal irradiation with curative intent. Overall survival was defined as the time from date of surgery to death or date of last follow-up and progression-free survival as the time from date of surgery to first event (progression or relapse) or date of last follow-up. Patients with follow-up time that exceeded 10 years were right-censored at 10 years.

The tightly defined age cutoff between the molecularly determined MB_SHH-Infant_ and MB_SHH-Child_ subgroups enabled us to assess an expanded survival cohort of MB_SHH-Child_ disease (n=55), including additional samples with insufficient DNA for methylation array analysis, classified as MB_SHH-Child_ on the basis of their age (appendix p 4). In this group, we assessed the prognostic potential of currently used clinical and molecular risk markers (M+ disease, R+ disease, LCA pathology, sex, *MYCN* amplification, *TERT* mutation, and *TP53* mutation [appendix pp 4–5]). Patients in the MB_SHH-Infant_ subgroup were typically younger than 3 years at diagnosis and were, therefore, treated on infant protocols. Treatment in this group of patients is heterogeneous, and is focused on omitting or delaying radiotherapy to reduce treatment-associated morbidities as far as possible. As a consequence, we report only overall survival in this group.

We created univariate and cross-validated multivariate Cox models based on subgroups, established risk factors, and cytogenetic changes. Prognostic markers in the multivariate analysis were identified by performing 100 rounds of 10-fold cross-validation, evaluating the performance of markers by measuring area under the curve (AUC) at 5 years for progression-free survival in the left out fold, and calculating the overall mean AUC over all rounds (appendix p 5). We added variables conferring an increase in AUC, as measured by time-dependent receiver operating characteristic curves at 5 years, to the model. Because MB_Grp3_ and MB_Grp4_ shared a metagene (V1), which defined a low-risk outcome and implied shared biology, we considered MB_Grp3/4_ as a single entity, and MB_Grp3_ and MB_Grp4_ separately for creation of survival models. In addition to currently understood clinical and molecular risk markers in these groups (M+ disease, R+ disease, LCA pathology, gender, *MYC*/*MYCN* amplification, and i17q [isochromosome 17q]), we additionally tested for recurrent cytogenetic changes, MB_Grp3_ membership, and membership of the high-risk methylomic group composed of members from both MB_Grp3_ and MB_Grp4_, defined by metagene V1 (appendix pp 5–6). We categorised identified independent prognostic markers into risk-stratification schemes (favourable-risk, >90% survival; standard-risk, >75–90% survival; high-risk, 40–75% survival; very high-risk, <40% survival) and survival-dependent ROC analysis of progression-free survival at 5 years, to assess performance^27^ by comparison with previously reported classification schemes (appendix pp 5–6).^16^, ^28^

We constructed Kaplan-Meier curves and compared patient groups with log-rank tests. For Kaplan-Meier comparison of two groups, we calculated hazard ratios (HRs) for the 0–5 year survival interval and 95% CIs from the Wald statistic. We tested the proportionality assumption for Cox modelling using scaled Schoenfeld residuals. Missing data were assumed to be missing completely at random and affected samples were removed from multivariate analyses. We implemented array processing, normalisation, quality-control checks, and copy-number estimation, relative to a panel of 18 normal cerebella with the R packages minfi^29^ and conumee (appendix p 2).

The significance threshold was set at p<0·05 for all statistical tests in this study, unless otherwise stated. Significance of association was assessed using Fisher's exact and chi-squared tests with Yates' continuity correction. We identified subgroup-specific age-differences between the non-MB_WNT_ or non-MB_SHH_ medulloblastoma subgroups using ANOVA (appendix p 4). Statistical or bioinformatics analyses were done using R (version 3.2.3).

### Role of the funding source

The funders of the study had no role in study design, data collection, data analysis, data interpretation, or writing of the report. The corresponding author had full access to all of the data and had the final responsibility to submit for publication.

## Results

**Figure 1:**
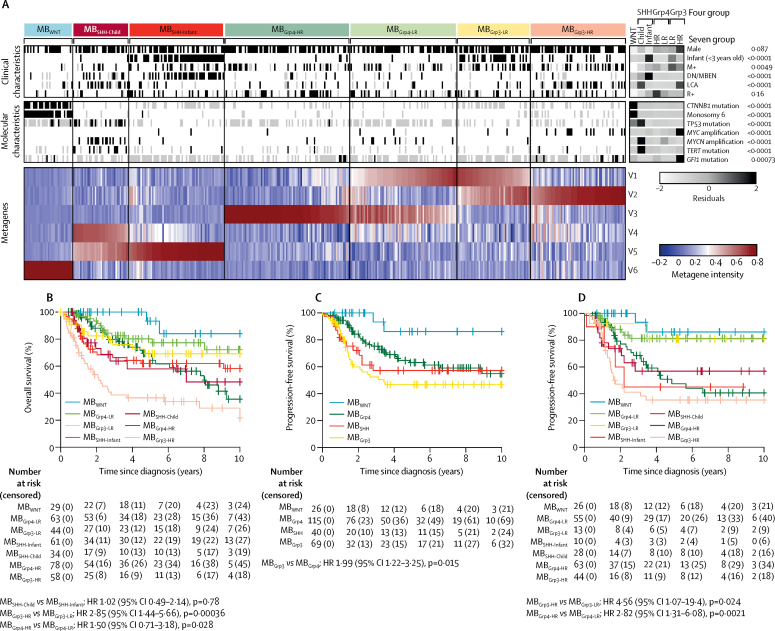
Novel clinically significant subgroups within the established medulloblastoma subgroups (A) Non-negative matrix factorisation consensus clustering of methylome data from 428 primary medulloblastomas. Each column represents one patient. Missing data are shown in grey. Residuals from χ^2^ tests indicate where subgroup-enrichment has occurred (darker shades of grey indicate stronger relationships), p values are from χ^2^ tests of enrichment; scale bar for residuals (−2 to 2) is shown. Methylation-derived metagene levels (V1–V6), which define subgroup membership, are also shown (red indicates high metagene levels, blue indicates low levels). (B) Overall survival of patients in the seven identified subgroups. All discovery cohort patients with available overall survival information are shown (n=367). (C) Progression-free survival of patients in the consensus four subgroups of medulloblastoma in discovery cohort patients receiving craniospinal irradiation and aged 3–16 years at diagnosis (n=250). (D) Progression-free survival of patients in the seven identified subgroups of medulloblastoma in patients receiving craniospinal irradiation and aged 3–16 years at diagnosis (n=239). Discrepancy in the numbers of patients in (C) and (D) is due to consensus clustering; certain samples could not be confidently classified for the seven subgroup model or the four subgroup model, and were omitted from the figures. DN/MBEN=desmoplastic or nodular medulloblastoma with extensive nodularity. HR=hazard ratio. LCA=large-cell anaplastic. M+=metastatic disease. R+=residual disease.

Clinicopathological and molecular diagnostic characteristics of 428 patients younger than 16 years who had primary childhood medulloblastoma (discovery cohort) are shown in table 1. Consensus analysis identified two equally robust cluster solutions (cophenetic index 0·998 [four metagenes] and 0·997 [six metagenes]; appendix p 10). The first cluster solution (four metagenes, four clusters) recapitulated the established four-subgroup consensus,^1^ whereas the second (six metagenes, seven clusters) revealed further clusters within the established subgroups (figure 1A, appendix pp 10–11).

**Table 1:** Demographics and clinicopathological characteristics of all cohorts

		**Discovery cohort (n=428)**	**Validation cohort (n=276)**	**MB_SHH-Child_ survival cohort (n=55)**	**MB_Grp3/4_ survival cohort (n=175)**
Sex					
	Male	278 (65%)	174 (63%)	32 (58%)	124 (71%)
	Female	150 (35%)	102 (37%)	23 (42%)	51 (29%)
	Male:female ratio	1·9:1	1·7:1	1·4:1	2·4:1
Age at diagnosis (years)					
	Median (range)	6·34 (0·24–15·97)	7·50 (0·0–18·0)	10·86 (3·5–15·54)	7·33 (3·4–15·97)
	<3	101 (24%)	30 (11%)	0	0
	≥3	327 (76%)	244 (89%)	55 (100%)	175 (100%)
Pathology variant					
	Classic	276 (70%)	NA	23 (44%)	131 (83%)
	DN/MBEN	58 (15%)	NA	15 (29%)	7 (5%)
	LCA	60 (15%)	NA	14 (27%)	19 (12%)
	MB-NOS	34	NA	3	18
Metastatic stage					
	M–	285 (73%)	NA	47 (85%)	124 (73%)
	M+	104 (27%)	NA	8 (15%)	47 (27%)
Resection					
	Sub-total resection (R+)	98 (26%)	NA	9 (16%)	51 (29%)
	Gross total resection (R–)	285 (74%)	NA	46 (84%)	123 (71%)
Treatment					
	RTX alone	28 (8%)	NA	16 (33%)	16 (9%)
	RTX and CTX	314 (92%)	NA	32 (67%)	157 (91%)
Follow-up time (years)		4·91 (0·2–25·7)	NA	6·52 (0·5–16·8)	4·58 (0·4–25·7)
*CTNNB1* mutation					
	Mutant	24 (7%)	NA	NA	0
	Wild-type	297 (93%)	NA	NA	144 (100%)
Chromosome 6					
	Loss	30 (8%)	28 (10%)	NA	0
	Normal	361 (92%)	248 (90%)	NA	158 (100%)
Chromosome 17					
	i17q	111 (28%)	87 (32%)	NA	72 (46%)
	No i17q	280 (72%)	189 (68%)	NA	86 (54%)
*MYC* amplification					
	Positive	22 (5%)	12 (4%)	NA	8 (5%)
	Negative	404 (95%)	264 (96%)	NA	165 (95%)
*MYCN* amplification					
	Positive	29 (7%)	17 (6%)	NA	11 (6%)
	Negative	397 (93%)	259 (94%)	NA	162 (94%)
*TP53* mutation					
	Positive	18 (7%)	NA	13 (27%)	1 (1%)
	Negative	238 (93%)	NA	35 (73%)	89 (99%)
*TERT* mutation					
	Positive	16 (4%)	NA	18 (35%)	1 (1%)
	Negative	357 (96%)	NA	34 (65%)	150 (99%)
450K 4 subgroup assignment					
	MB_WNT_	33 (8%)	33 (12%)	NA	0
	MB_SHH_	109 (26%)	60 (22%)	24 (100%)	1 (1%)
	MB_Grp3_	130 (31%)	72 (26%)	NA	63 (36%)
	MB_Grp4_	153 (36%)	111 (40%)	NA	109 (63%)
	Non-classifiable	3	NA	NA	2
450K 7 subgroup assignment					
	MB_WNT_	33 (8%)	33 (12%)	NA	NA
	MB_SHH-Child_	38 (9%)	32 (12%)	24 (100%)	NA
	MB_SHH-Infant_	65 (16%)	28 (10%)	NA	NA
	MB_Grp3-HighRisk_	65 (16%)	51 (18%)	NA	44 (25%)
	MB_Grp3-LowRisk_	50 (12%)	20 (7%)	NA	13 (7%)
	MB_Grp4-HighRisk_	85 (21%)	54 (20%)	NA	63 (36%)
	MB_Grp4-LowRisk_	73 (18%)	58 (21%)	NA	55 (31%)
	Non-classifiable	19	0	NA	NA

Data are n (%) or median (range), unless otherwise specified. MB=medulloblastoma. SHH=sonic hedgehog. Grp3=group 3. Grp4=group 4. Grp3/4=combined groups 3 and 4. NA=data not available. DN=desmoplastic or nodular. MBEN=medulloblastoma with extensive nodularity. LCA=large-cell anaplastic. MB-NOS=medulloblastoma not otherwise specified. M–=non-metastatic disease. M+=metastatic disease. RTX=radiotherapy. CTX=chemotherapy. WNT=wnt/wingless.

MB_WNT_ tumours formed a single subgroup (n=33) characterised by *CTNNB1* mutations, loss of chromosome 6, and an expected favourable prognosis (5-year overall survival: 93% [95% CI 82–100]; figure 1B). Our newly detected metagenes split each remaining consensus subgroup (MB_SHH_, MB_Grp3_, and MB_Grp4_) in two. MB_SHH_ was split into age-dependent subgroups corresponding to infant (<4·3 years; MB_SHH-Infant_; n=65) and childhood patients (≥4·3 years; MB_SHH-Child_; n=38) by the respective absence or presence of metagene V4. Both have intermediate prognoses (5-year overall survival MB_SHH-Child_: 58% [95% CI 41–82]; MB_SHH-Infant_: 62% [50–77]; figure 1B). MB_Grp3_ and MB_Grp4_ are each split into high-risk (MB_Grp3-HR_ [n=65] and MB_Grp4-HR_ [n=85]) and low-risk (MB_Grp3-LR_ [n=50] and MB_Grp4-LR_ [n=73]) subgroups by common metagene V1 (figure 1A). 5-year overall survival was 37% [95% CI 25–53] in the MB_Grp3-HR_ subgroup, 69% [55–87] in the MB_Grp3-LR_ subgroup, 69% [58–83] in the MB_Grp4-HR_ subgroup, and 80% [70–92] in the MB_Grp4-LR_ subgroup (figure 1B). The subdivision of MB_Grp3_ and MB_Grp4_ distinguishes patients with a superior stratification (5-year overall survival AUC 0·649 [MB_Grp3/4_ combined with low-risk or high-risk subdivision]) compared with the current consensus MB_Grp3_ and MB_Grp4_ subgroups (AUC 0·610). Moreover, in the patients aged 3–16 years at diagnosis and receiving craniospinal irradiation, the high-risk or low-risk subdivision of MB_Grp3/4_ stratifies this group into standard (MB_Grp3-LR_ 81% [95% CI 60–100%]; MB_Grp4-LR_ 81% [71–93%]) and high-risk (MB_Grp3-HR_ 35% [23–55%]; MB_Grp4-HR_ 47% [34–66%]) 5-year progression-free survival outcomes, by contrast with the current consensus MB_Grp3/4_ designations, which show intermediate outcomes (figure 1C, 1D).

Clinicopathological and biological features were non-randomly distributed in all seven subgroups (figure 1A, appendix pp 12–15). Patients in the MB_SHH-Infant_ subgroup had significantly enriched desmoplastic or nodular pathology compared with all other subgroups (p<0·0001), and *TP53* mutation (p<0·0001) and *MYCN* amplifications (p<0·0001) were significantly more frequent in MB_SHH-Child_ than in all other subgroups. Patients in the MB_Grp3-HR_ subgroup significantly more frequently had LCA pathology (p<0·0001) and *MYC* amplification (p<0·0001), than all other subgroups. Although patients in the MB_Grp3-HR_ and MB_Grp4-HR_ subgroups had similar 10-year overall survival (22% [95% CI 10–46] *vs* 36% [22–59]; figure 1B), patients in the MB_Grp4-HR_ subgroup died later of their disease (ten [36%] of 28 deaths in the MB_Grp4-HR_ subgroup occurred more than 5 years after diagnosis) than did those in the MB_Grp3-HR_ subgroup (33 [92%] of 36 deaths occurred within 5 years of diagnosis; appendix p 26).

Validation by projection of six metagenes onto an independent cohort^8^ of 276 patients (table 1) confirmed their existence (appendix pp 10–11). Moreover, reapplying consensus clustering to the combined cohort of 704 patients confirmed a seven subgroup model as optimal, giving 100% concordance to the classifications derived separately from our discovery cohort (appendix pp 10–11).

**Figure 2:**
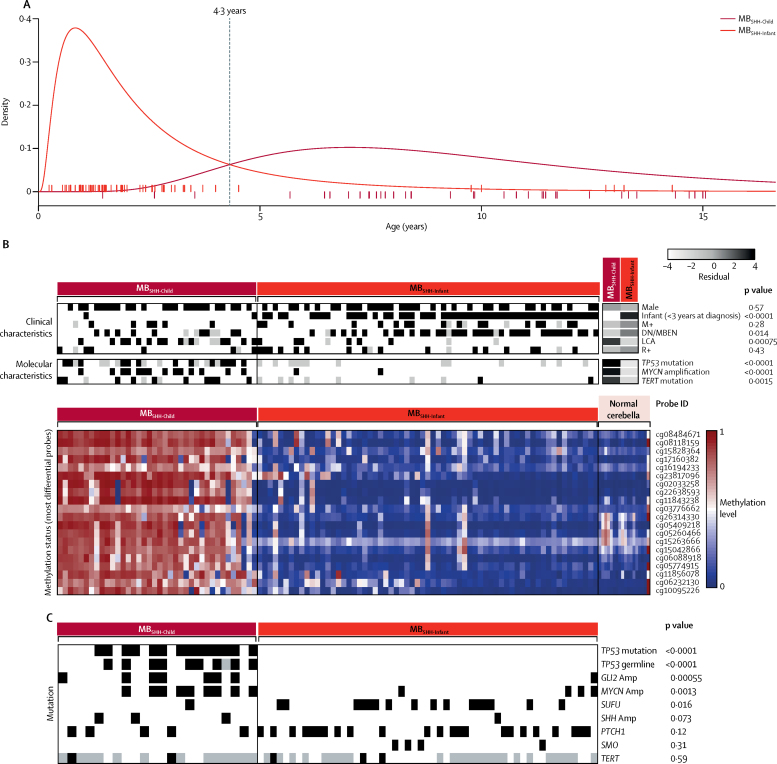
MB_SHH_ disease comprises two age-dependent molecular subgroups (A) Log-normal age distributions of MB_SHH-Infant_ (red) and MB_SHH-Child_ disease (dark red). Patient ages at diagnosis are shown as ticks along the x-axis and are coloured by subgroup. (B) Clinicopathological and molecular disease features of MB_SHH-Infant_ and MB_SHH-Child_ subgroups. Residuals from χ^2^ tests indicate where subgroup-enrichment has occurred (darker shades of grey indicate stronger relationships); scale bar for residuals (−4 to 4) is shown. p values from χ^2^ tests are shown. Differentially methylated probes: Illumina probe identifiers for the top 20 most differentially methylated probes, alongside methylation status of 18 normal cerebella (pink). Each column represents one patient. (C) SHH genome-sequencing data^26^ was classified into methylation subgroups on the basis of age. Each column represents one patient. Amp=amplification. DN/MBEN=desmoplastic or nodular medulloblastoma with extensive nodularity. LCA=large-cell anaplastic. M+=metastatic disease. R+=residual disease.

Age distributions differed between the two MB_SHH_ subgroups; age distributions are log-normally distributed and intersect at 4·3 years (figure 2A). The two peak incidences of age at diagnosis in infants and in older children for MB_SHH_ disease,^26^ when observed as a whole, are resolved by their classification into distinct MB_SHH-Infant_ and MB_SHH-Child_ subgroups (appendix pp 12–13). Each MB_SHH_ subgroup possesses characteristic molecular or clinicopathological features (appendix pp 12–13). LCA pathology (p=0·00050), *MYCN* amplification (p<0·0001), and mutations of *TP53* (p<0·0001) and *TERT* (p=0·0015) were all significantly enriched in the MB_SHH-Child_ subgroup compared with the MB_SHH-Infant_ subgroup; whereas gender, M+ disease status, and R+ disease status were not significantly different between groups (figure 2B; appendix pp 12–13). *TERT* promoter mutation and *MYCN* amplification or LCA pathology were mutually exclusive (figure 2B; appendix pp 12–13). Mutational data from an independent MB_SHH_ cohort^26^ showed that *SUFU* mutation was significantly associated with MB_SHH-Infant_, whereas *PTCH1* mutations were observed in both MB_SHH_ subgroups (figure 2C). *GLI2* amplification, *MYCN* amplification, and *TP53* mutations (both somatic and germline) were significantly associated with the MB_SHH-Child_ subgroup (figure 2C).

Compared with normal cerebella and patients in the MB_SHH-Infant_ subgroup, patients in the MB_SHH-Child_ subgroup had subgroup-specific DNA methylation changes (predominantly hypermethylation), at both individual CpG loci and at the gene level (figure 2B; appendix pp 12–13), frequently involving developmental genes (79 [14%] of 584 genes with gene ontology term embryonic morphogenesis had aberrant hypermethylation). DNA methylation changes were validated in an independent cohort^8^ (appendix pp 12–13). When discovery cohort MB_SHH_ RNA-seq expression data were available (190 [44%] of 428 samples), significant differential expression was observed between the subgroups (1593 genes, fold change >1·5; adjusted p<0·01; appendix pp 12–13). Although there were few recurrent cytogenetic changes, many tumours in patients in the MB_SHH-Child_ subgroup (18 [51%] of 35 tumours) had loss of chromosome 9q, often associated with gain of 9p (appendix pp 12–13).

**Figure 3:**
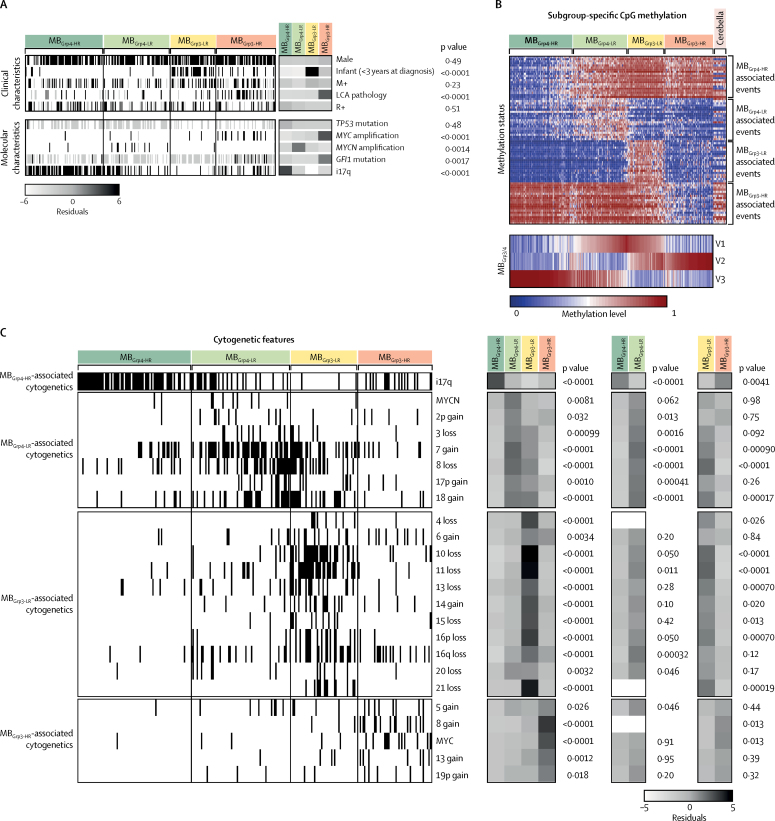
Characterisation of MB_Grp3_ and MB_Grp4_ subgroups (A) Clinicopathological and molecular disease features. Residuals from χ^2^ tests indicate where subgroup-enrichment has occurred (darker shades of grey indicate stronger relationships); scale bar for residuals (−6 to 6) is shown. p values from χ^2^ tests are shown. (B) Heat map shows the top 20 differentially methylated probes for these subgroups. Methylation data of 18 normal cerebella are shown alongside and magnitude of MB_Grp3_ and MB_Grp4_ metagenes is shown below. (C) Identification of MB_Grp3_ and MB_Grp4_ medulloblastoma cytogenetic determinants. Markers with p<0·05 and present in at least 10% of one subgroup are ordered by their subgroup association and then by chromosomal order. Residuals from χ^2^ tests indicate where subgroup enrichment has occurred (darker shades of grey indicate stronger relationships), across all subgroups and within MB_Grp3_ and MB_Grp4_ individually. p values from χ^2^ tests are shown. i17q=isochromosome 17q. LCA=large-cell anaplastic. M+=metastatic disease. R+=residual disease.

The age distributions of patients in the four MB_Grp3_ and MB_Grp4_ subgroups differed significantly (p<0·0001). Patients in the MB_Grp3-LR_ and MB_Grp3-HR_ subgroups were younger at diagnosis than those in the MB_Grp4-LR_ and MB_Grp4-HR_ subgroups (appendix pp 14–15). Infants in the MB_Grp3-HR_ subgroup frequently had amplified *MYC* (seven [64%] of 11 infants). MB_Grp3-HR_ tumours were strongly associated with LCA pathology (20 [35%] of 57) and *GFI1* mutations (nine [29%] of 31; figure 3A, appendix pp 14–15). i17q is the sole significantly enriched feature of MB_Grp4-HR_ (60 [76%] of 79 [figures 3A, 3C]. Clinicopathological and molecular disease features of the MB_Grp3_ and MB_Grp4_ subgroups are shown in figure 3A.

Several hundred differentially methylated CpG probes or regions defined the four subgroups. MB_Grp3-HR_ was characterised by the greatest number of significantly differentially methylated CpGs compared with other subgroups, commonly hypomethylated CpG loci (figure 3B; appendix pp 14–15). Notably, the low-risk subgroups were defined primarily by hypermethylation with respect to normal cerebellum, whereas the high-risk subgroups were defined by hypomethylation (figure 3B; appendix pp 14–15). Cytogenetic changes distinguished each subgroup as unique from the others (figure 3C). These distinguishing cytogenetic features were validated in an independent cohort (appendix pp 14–15).

We did survival analyses in an MB_SHH-Child_ cohort that included 31 additional SHH cases unsuitable for 450k array analysis and classified as MB_SHH-Child_ on the basis of age (appendix pp 4–5). In this cohort, one out of three assessable *TP53* mutations were germline (appendix pp 16–17). *TP53* mutation was significantly associated with *MYCN* amplification (p=0·022) and LCA pathology (p=0·0033), *MYCN* amplification was associated with LCA pathology (p<0·0001), and LCA pathology and *MYCN* amplification were never observed with *TERT* mutations (p=0·00079 for LCA and p=0·0090 for *MYCN* amplification). There was no significant association between metastatic (M+) disease and *TP53* mutation (p=1), *MYCN* amplification (p=0.15), or LCA pathology (p=0·67), or an association between sub-totally resected (R+) disease and *TP53* mutation (p=1), *MYCN* amplification (p=1) or LCA pathology (p=0·41). Univariate survival analysis of clinicobiological features (including risk features established in disease-wide studies^16^) in this cohort showed significantly shorter progression-free survival associated with *MYCN* amplification *TP53* mutation, LCA pathology, M+ disease, and R+ disease, but no associations with *TERT* mutation status or sex (table 2; appendix pp 18–19). Multivariate Cox modelling, showed that *MYCN* amplification, *TP53* mutation, and M+ disease are independent risk factors for progression-free survival (table 2). Only the 42 samples with complete clinical information for the considered variables were included. The disease-wide risk-stratification scheme currently in use for the HIT-SIOP-PNET5-MB clinical trial,^16^ which deems *MYCN* amplification, LCA pathology, M+ disease, and R+ disease as high-risk factors, outperformed the MB_SHH-Child_ subgroup stratification in AUC analysis (appendix pp 16–17). We used this HIT-SIOP-PNET5-MB stratification scheme as the basis of a combined risk-stratification model for MB_SHH-Child_ (appendix pp 16–17), classifying patients with any one of these risk factors as very high risk. 50 patients had sufficient clinical data for classification using the scheme. This model discriminates favourable (24 [48%] of 50 patients, 5-year progression-free survival: 96% [95% CI 88–100]) and very high-risk (26 (52%), 5-year progression-free survival: 29% [14–58]) groups of patients within the MB_SHH-Child_ subgroup (p<0·0001; appendix pp 16–17).

**Table 2:** Identification of prognostic survival markers in MB_SHH-Child_ cohort

	**n**	**Univariate (n=55)**		**Cross-validated multivariate (n=42)**	
		HR (95% CI)	p value	HR (95% CI)	p value
*MYCN* amplification *vs* no amplification	52	4·47 (1·65–12·1)	0·0032	2·83 (0·87–9·22)	0·084
M+ *vs* M– disease	55	5·69 (2·01–16·0)	0·0011	4·59 (1·28–16·4)	0·019
*TP53* mutation *vs* no mutation	48	3·47 (1·29–9·30)	0·014	3·44 (1·15–10·2)	0·027
LCA pathology *vs* non-LCA pathology	52	2·88 (1·15–7·24)	0·025	..	..
*TERT* wild-type *vs TERT* mutation	52	2·21 (0·78–6·25)	0·13	..	..
R+ *vs* R– disease	55	3·45 (1·30–9·19)	0·013	..	..
Male *vs* female	55	1·13 (0·45–2·82)	0·79	..	..

p values are from Cox proportional hazards analyses. The prognostic significance of covariates selected in cross-validated multivariate models are also shown. HR=hazard ratio. M+=metastatic disease. M–=non-metastatic disease. LCA=large-cell anaplastic. R+=residual disease (subtotal surgical resection). R–=no residual disease (gross total resection).

Combining all craniospinally irradiated patients in the MB_Grp3/4_ subgroup aged 3–16 years who had outcome data (n=175), allocation to the MB_Grp3-HR_ and MB_Grp4-HR_ subgroups was a significant high-risk factor for shorter progression-free survival in univariate analysis (table 3). Additionally, in multivariate analysis, *MYC* amplification was identified as an independently prognostic high-risk factor, and chromosome 13 loss was associated with an improved outcome (table 3).

**Table 3:** Identification of prognostic survival markers in MB_Grp3_ and MB_Grp4_ cohorts

	**n**	**Univariate (n=175)**		**Cross-validated multivariate (n=133)**	
		HR (95% CI)	p value	HR (95% CI)	p value
High-risk methylation group *vs* low-risk methylation group	175	3·73 (1·94–7·18)	<0·0001	3·21 (1·59–6·51)	0·0012
*MYC* amplification *vs* no amplification	173	2·94 (1·06–8·13)	0·038	18·4 (5·01–67·7)	<0·0001
Loss of chromosome 13 *vs* no loss	158	0·10 (0·01–0·74)	0·024	0·06 (0·01–0·49)	0·0090
MB_Grp3_*vs* MB_Grp4_	175	2·04 (1·23–3·40)	0·006	..	..
M+ *vs* M– disease	171	1·77 (1·03–3·05)	0·039	..	..
i17q *vs* no i17q	158	1·71 (0·99–2·95)	0·056	..	..
Male *vs* female	175	1·56 (0·86–2·84)	0·144	..	..
*MYCN* amplification *vs* no amplification	173	0·72 (0·23–2·29)	0·576	..	..
LCA pathology *vs* non-LCA pathology	157	1·08 (0·49–2·39)	0·848	..	..
R+ *vs* R– disease	171	1·22 (0·72–2·09)	0·464	..	..

Identification of prognostic survival markers in combined childhood non-MB_SHH_ and non-MB_WNT_ survival cohort (aged 3·0–16·0 years, receiving craniospinal irradiation, with survival information). p values from Cox proportional hazards analyses are shown. The characteristics of covariates selected in cross-validated multivariate models are also shown. The high-risk methylomic group comprised samples from both MB_Grp3_ and MB_Grp4_, defined by the shared MB_Grp3/4_ metagene V1. HR=hazard ratio. MB=medulloblastoma. Grp3=group 3. Grp4=group 4. M+=metastatic disease. M–=non-metastatic disease. i17q=isochromosome 17q. LCA=large-cell anaplastic. R+=residual disease (subtotal surgical resection). R–=no residual disease (gross total resection).

**Figure 4:**
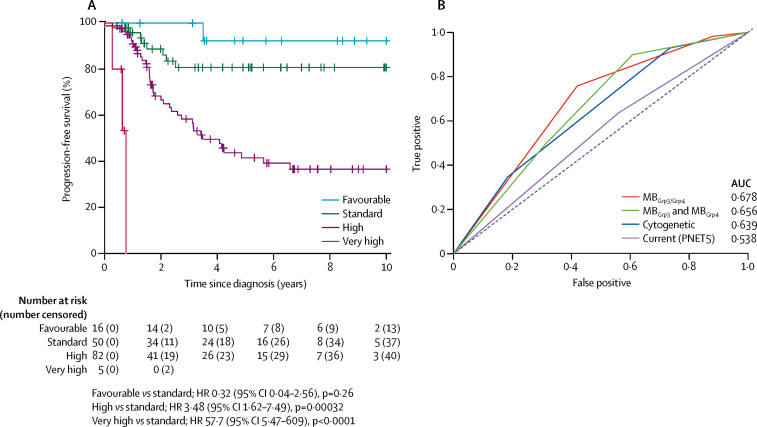
Novel risk stratification scheme for MB_Grp3_ and MB_Grp4_ medulloblastoma (A) Progression-free survival plots for identified risk subgroups (n=156) defined in table 3 and the appendix (p 20). (B) Time-dependent ROC curves at 5 years are shown for this novel risk stratification alongside a published cytogenetic stratification scheme^28^ (MB_Grp4_ with chromosome 11 loss or chromosome 17 gain, low risk; MB_Grp4_ with M– disease, standard risk; MB_Grp4_ with M+ disease, high risk; MB_Grp3_ with *MYC* amplification, i17q, or M+ disease, high risk; MB_Grp3_ without *MYC* amplification, i17q, or M+ disease, standard risk), and the PNET5 risk stratification (patients positive for one or more of LCA pathology, M+ disease, R+ disease, *MYC(N)* amplification are high risk; patients absent for all high-risk features, standard risk), as well as the stratification derived from considering MB_Grp3_ and MB_Grp4_ as separate entities (appendix p 22). AUC=area under curve. LCA=large-cell anaplastic. M+=metastatic disease. M–=non-metastatic disease. ROC=receiver operating characteristic.

A stratification model was developed that divided MB_Grp3/4_ into different risk groups for 5-year progression-free survival: favourable risk (chromosome 13 loss and no *MYC* amplification; 16 [10%] of 153 patients; 92% [95% CI 79–100]); standard risk (MB_Grp4-LR_ or MB_Grp3-LR_ with no *MYC* amplification; 50 [33%] patients; 81% [70–94]); high risk (MB_Grp4-HR_ or MB_Grp3-HR_ with no *MYC* amplification; 82 [54%] patients; 42% [31–56]); and very high risk (MB_Grp3_ with *MYC* amplification; five [3%] patients; 0%; figure 4A; appendix pp 20–21). 156 patients had information for chromosome 13 loss and *MYC* amplification, of which three were classed as unassignable because they were MB_Grp4_ with *MYC* amplification (appendix pp 20–21). This stratification scheme outperformed current risk-stratification models (figure 4B).

For comparison, we developed equivalent separate survival stratification schemes for MB_Grp3_ and MB_Grp4_ (appendix pp 22–23). Risk factors identified were broadly consistent with the factors identified in the combined scheme, although the combined scheme was a better predictor of progression-free survival than when MB_Grp3_ and MB_Grp4_ were considered separately (figure 4B). Taking MB_Grp4_ patients in isolation, in univariate analysis, a designation of MB_Grp4-HR_, chromosome 7q status, M+ disease, and male sex were associated with poor progression-free survival, whereas *MYCN* amplification, R+ disease, and LCA pathology were not (appendix pp 22–23). Chromosome 7q gain and M+ disease were retained as independent prognostic factors in multivariate analysis (appendix pp 22–23). A 5-year progression-free survival model incorporating chromosome 7q gain and M+ disease defined standard-risk (35 [32%] of 110 patients; 87% [95% CI 76–100]) and high-risk groups (75 [68]; 49% [37–66]), and outperformed other published models by AUC analysis (appendix pp 22–23).

Taking patients with MB_Grp3_ in isolation, *MYC* amplification was the only risk factor significantly associated with progression-free survival in multivariate analysis, and outcomes were poor for these very high-risk patients (appendix pp 22–23). Patients in the MB_Grp3_ with non-*MYC* amplified tumours were at high risk, with progression-free survival similar to that for the MB_Grp4-HR_ subgroup (51 [91%] of 56 patients; 46% [95% CI 33–64] for MB_Grp3_ with non-*MYC* amplified tumours *vs* 41% [28–60] for MB_Grp4-HR_). MB_Grp3-HR_ shows a worse outcome than MB_Grp3-LR_ (p=0·040; appendix p 22). LCA pathology (11 [21%] of 53 patients), M+ disease (17 [29%] of 58 patients), and R+ disease (13 [22%] of 59 patients),^1^ were frequent in patients in MB_Grp3_ but none were associated with prognosis, and no stratification scheme based on MB_Grp3_ alone markedly improved outcome prediction compared with standard stratification schemes (appendix pp 22–23).

**Figure 5:**
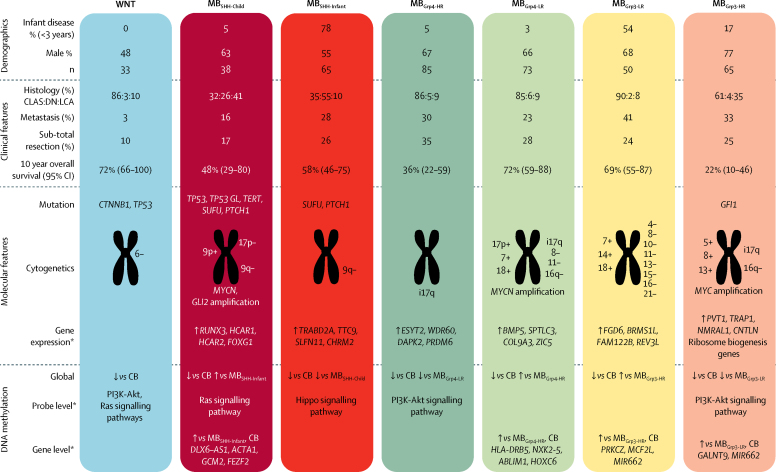
Summary of the seven primary childhood medulloblastoma subgroups Demographic, clinicopathological, and molecular features are summarised. *Comparisons of cytogenetic, gene expression, and DNA methylation changes are made with respect to their counterpart subgroup, except for MB_WNT_ cases, which were compared with normal cerebella if data were available. For probe-level comparisons, Kyoto Encyclopedia of Genes and Genomes pathway enrichment of demethylated loci was investigated, after correcting for multiple probes mapping to the same gene (data summarised in appendix pp 27–31). CB=normal cerebella. CLAS=classic histological subtype. DN=desmoplastic nodular. LCA=large-cell anaplastic.

**Figure 6:**
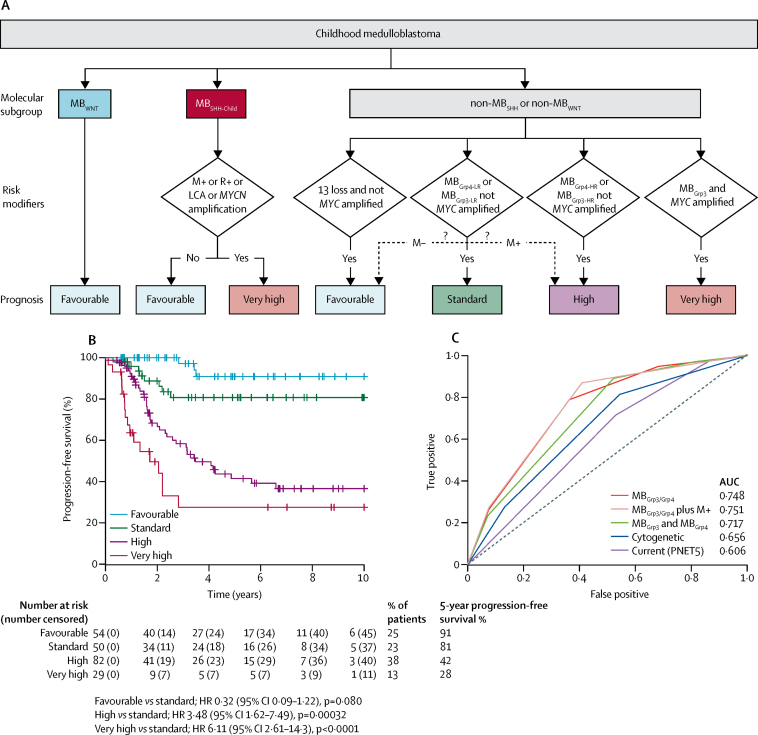
Summary of survival modelling of novel medulloblastoma subgroups (A) Summary of a novel risk-stratification scheme for childhood medulloblastoma in a cohort of patients aged 3–16 years receiving craniospinal irradiation (n=215). The potential to further stratify MB_Grp4-LR_ patients into favourable and high-risk groups by their metastatic stage is shown (dashed arrows). (B) Kaplan-Meier plot of childhood medulloblastoma risk stratification. (C) Performance of novel stratification scheme in comparison with time-dependent ROC curves of existing schemes of progression-free survival at 5 years. MB_Grp3/4_: MB_Grp3_ and MB_Grp4_ considered as a single entity; MB_Grp3/4_ plus M+: MB_Grp3_ and MB_Grp4_ considered as a single entity with MB_Grp4-LR_ and non-*MYC* amplified MB_Grp3-LR_ further stratified by M+ disease status; MB_Grp3_ and MB_Grp4_: MB_Grp3_ and MB_Grp4_ stratified separately; cytogenetic: cytogenetically defined scheme;^28^ PNET5: scheme employed by HIT-SIOP-PNET5-MB clinical trial. LCA=large-cell anaplastic. M+=metastatic disease. M–=non-metastatic disease. R+=residual disease.

The clinicopathological and molecular features of the new seven clinically significant subgroups are summarised in figure 5. The combination of subgroup-specific survival models creates an overarching risk stratification for all childhood medulloblastoma (figure 6A). Patients are stratified into four clinical risk groups for 5-year progression-free survival: favourable risk (comprising MB_WNT_, MB_SHH-Child_ with no high-risk features, and non-*MYC* amplified MB_Grp3/Grp4_ with chromosome 13 loss; 54 [25%] of 215 patients; 91% [95% CI 82–100]); standard risk (comprising non-*MYC* amplified MB_Grp3-LR/Grp4-LR_ subgroups; 50 [23%] patients; 81% [70–94]); high-risk (comprising non-*MYC* amplified MB_Grp3-HR/Grp4-HR_ subgroups; 82 [38%] patients; 42% [31–56]); and very high-risk (comprising MB_SHH-Child_ with high-risk features and *MYC*-amplified MB_Grp3_; 29 [13%] patients; 28% [14–56]; figure 6B). 215 patients aged 3–16 years at diagnosis had data available for these factors. The AUC from our proposed stratification of childhood medulloblastoma outperforms current and proposed cytogenetic risk stratifications (figure 6C).^28^ We note that M+ disease status is a strong risk factor for poor progression-free survival in MB_Grp4_. Incorporation of M+ disease status into MB_Grp4-LR_ and non-*MYC* amplified MB_Grp3-LR_ survival modelling does not affect model performance, but potentially allows redistribution of standard-risk patients to create larger favourable (90 [41%] of 218 patients) and high-risk groups (99 [45%] of 218 patients; figure 6A, C; appendix pp 24–25), which could be considered as an alternative stratification scheme. The proposed refinement to the stratification enables additional cases classified as MB_Grp3-LR_ and MB_Grp4-LR_ that do not have copy number information (other than *MYC* amplification status) and are non-metastatic to be classified as favourable.

## Discussion

The discovery and validation of seven robust and reproducible primary molecular subgroups of childhood medulloblastoma in this retrospective cohort study represents, to our knowledge, the first clinically significant elaboration of the four-subgroup consensus established in 2012.^1^ While our work supports the stability of the four established groups, it also reveals significant substructures within each group with distinct clinicopathological and molecular features. Importantly, these primary subgroups emerge from unsupervised analysis, and are supported by distinguishing DNA methylation, gene expression, and copy-number profiles, consistent in discovery and validation cohorts. Notably, these subgroups were not identifiable in a previously published dataset, which included fewer samples and, specifically, fewer infant patients.^8^ Our seven subgroups reveal a biological overlap between MB_Grp3_ and MB_Grp4_. They share a biological signature, defined by a common metagene, indicating a clinicobiological overlap, which might suggest a common origin.

These primary subgroups may be further subdivided by the presence or absence of secondary molecular characteristics, many of which, in turn, have subgroup-specific clinical and prognostic significance (eg, *MYC* amplification in MB_Grp3_ or *TP53* mutation, *MYCN* amplification, LCA pathology, M+ disease, and R+ disease in MB_SHH-Child_). Some of these secondary features have been described and assigned clinical significance in previous studies; in this Article, their association with specific novel subgroups (eg, chromosome 11 loss and chromosome 17 gain in MB_Grp4-LR_^28^) has revealed the underlying biological basis of these subgroup-specific biomarkers. Moreover, re-evaluation of currently used high-risk factors derived from cohort-wide studies that did not consider subgroup shows that their importance is either low (eg, LCA pathology, M+ disease, or R+ disease in MB_Grp3_; *MYCN* in MB_Grp4_) or high (*MYCN* amplification, LCA pathology, *TP53* mutation, and M+ disease in MB_SHH-Child_; *MYC* in MB_Grp3_; M+ in MB_Grp4_) when considered in the context of our new subgroups. Finally, the biological definition of MB_SHH-Infant_ (<4·3 years) is at odds with current clinical definitions of infant disease (<3 years) and this should prompt consideration in the future as to whether infant treatment protocols are appropriate for MB_SHH-Infant_ patients older than 3 years.^16^ Survival modelling in children younger than 3 years is qualitatively different from analysis in those over 3 years of age, because of the heterogeneity of treatment of infant disease. As such, we regarded further risk modelling in this patient group to be outside the scope of this study, to be addressed in future investigations. To our knowledge, no previous study has directly assessed survival of the molecularly-defined MB_SHH-Infant_ subgroup. The overall survival at 5 years that we observed in MB_SHH-Infant_ disease (62%, 95% CI 50–77) is lower than previously reported in an international meta-analysis of the MB_SHH_ subgroup in age-defined infants (<4 years at diagnosis; 77%),^30^ but these patients were not molecularly defined and, as such, are not directly comparable.

Our survival analysis focused on the 3–16-year-old clinical group who received current conventional therapies: surgical resection followed by adjuvant radiotherapy with or without chemotherapy at diagnosis with curative intent. Combined risk-modelling across all patients in the non-MB_WNT_ or non-MB_SHH_ subgroups identified *MYC* amplification, high-risk methylation subgroup membership, and loss of chromosome 13 as independent risk factors. Survival models incorporating these factors outperformed the clinical risk-stratification used in current clinical trials (HIT-SIOP-PNET5-MB^16^) and subgroup-dependent cytogenetic stratification schemes.^28^

We have defined a risk-stratification of childhood medulloblastoma that allows patients to be assigned into four overarching risk groups. Favourable-risk patients, including both MB_WNT_ and novel non-MB_WNT_ groups, should be urgently considered for therapy-reducing strategies. Very high-risk patients, typically refractory to conventional therapies (eg, amplified *MYCN*, mutated *TP53*, LCA pathology, and M+ disease in MB_SHH_ and amplified *MYC* in MB_Grp3_) should be prioritised for alternative upfront treatment strategies. The priority for high-risk patients, comprising the novel MB_Grp4-HR_ and patients with non-amplified *MYC* in the MB_Grp3-HR_ subgroup, and a standard-risk group, comprising all other patients, should be optimisation of current therapies and the application of novel, biologically targeted agents.

We note the limitations of developing survival models in retrospective patient cohorts, who received heterogeneous treatments. Notwithstanding that models were developed using patients aged 3–16 years, who all received maximal surgical resection and craniospinal irradiation with curative intent, caution should be applied to their clinical implementation. We also note the statistical limitations of stratifications identifying small numbers of patients (eg, very high-risk, 13% of patients). Moreover, some of the identified biomarkers (notably loss of chromosome 13) have not previously been reported as prognostic. We therefore emphasise that validation in additional cohorts, and ideally in prospective, uniformly treated patients in clinical trials, is essential. A small number of samples (<5 samples) from this study were used to assist with the creation of the four-subgroup classification consensus.^5^ Similarly, our own publication that described four methylation-dependent subgroups of medulloblastoma^7^ contained 87 samples that overlapped with this study, although the previously published study contained fewer samples (discovery cohort size of 100 and validation cohort size of 130 patients) and DNA methylation profiling was at much lower resolution (1505 *vs* >400 000 CpG loci).

The existence of novel primary medulloblastoma subgroups, coupled with the characterisation of secondary prognostic features within each group, represents a significant advance in our understanding of medulloblastoma biology and its application in clinical management and future trials design. We provide clear evidence of the shared biology between MB_Grp3_ and MB_Grp4_, which affects clinical behaviour and has significant implications for understanding disease biology, developmental origins, and experimental modelling. These investigations constitute a blueprint for a new consensus in medulloblastoma molecular sub-classification with important implications for future molecular diagnostics and clinical management.
