# Supplementary material for: Novel molecular subgroups for clinical classification and outcome prediction in childhood medulloblastoma: a cohort study
Source: Lancet Oncol. 2017 Jul;18(7):958–71. doi: 10.1016/S1470-2045(17)30243-7 (PMC5489698; doi:10.1016/S1470-2045(17)30243-7)
Supplement: Supplementary appendix [file mmc1.pdf]

THE LANCET
Oncology

Supplementary appendix

This appendix formed part of the original submission and has been peer reviewed. We post it as supplied by the authors.

Supplement to: Schwalbe EC, Lindsey JC, Nakjang S, et al. Novel molecular subgroups for clinical classification and outcome prediction in childhood medulloblastoma: a cohort study. *Lancet Oncol* 2017; published online May 22. http://dx.doi.org/10.1016/S1470-2045(17)30243-7.

Appendix: Novel molecular subgroups for clinical classification and outcome prediction in childhood medulloblastoma: a cohort study.

Table of Contents

Supplementary Methods 2

Methylation array processing 2

Subgroup identification and validation 2

Subgroup characterisation 3

Transcriptome analysis 4

Mutational analysis 4

Genomic copy number specific analysis 4

Survival analysis 4

Supplementary Methods References 7

Supplementary Figures and Tables 9

Supplementary Methods

Methylation array processing

DNA extracts from a cohort of 428 centrally-reviewed, clinically-annotated primary medulloblastomas from patients aged 0-16·0 years at diagnosis, collected predominantly from UK Children's Cancer and Leukaemia Group (CCLG) (n=366/428; 86%), alongside samples from the UKCCSG-SIOP-PNET3 clinical trial (n=27/428; 6%) and from collaborating European Institutions in Budapest (n=20/428; 5%) and Warsaw (n=15/428; 4%) were run on Illumina 450k DNA methylation microarrays. The cohort consisted of 302 (71%) medulloblastomas extracted from fresh-frozen material, and 126 (29%) from formalin-fixed, paraffin-embedded (FFPE) tumours. This cohort was enriched for infant (aged <3 at diagnosis) cases (n=101/428 (24%)), since we hypothesised that the methylome of infant disease was substantially different from childhood disease. Cohort demographics of this centrally-reviewed and comprehensively clinically annotated cohort are summarised in Table 1. Of note, 87 cases from this cohort overlapped with our previous study which identified 4 methylation-dependent subgroups of medulloblastoma using low-resolution methylation arrays[1].

Methylation array processing, normalisation and quality control checks were implemented using the R package minfi[2]. We employed conservative quality control measures to filter out poorly performing and potentially confounding loci: Probes mapping to the sex chromosomes, genotyping probes, probes aligning to more than one place in the genome, and probes which had a SNP with a minor allele frequency of 5% or greater within 50bp of the interrogated site were removed. Probes with a detection p value > 0·05 in more than 5% of samples were removed[3]. Remaining probes with a detection p value > 0·05 were imputed using k-nearest neighbour imputation, as previously described[4]. Samples were checked for potential duplication by examining pairwise correlations of 65 genotyping probes.

Subsequently, using 18 normal cerebellum samples as controls (consisting of 3 foetal, 10 infant (newborn-25 months) and 3 adult samples (43-67 years), with 2 samples of unknown age; all samples consisted of post-mortem material from patients who died from non-neoplastic events), copy number profiles were generated from 450k methylation array probe intensity values using the R package conumee (https://www.bioconductor.org/packages/release/bioc/html/conumee.html; date accessed Mar 2nd, 2017), and arm-level genomic copy-number gain and loss was scored. Using copy number information derived from gold-standard Affymetrix SNP6·0 arrays, cutoffs for calling chromosomal arm gain (log ratio > 0·12) and loss (log ratio < -0·22) were empirically derived by using confirmed monosomy of chromosome 6 in MBWNT tumours as a marker of chromosomal loss, and confirmed gain of chromosome 7, the most commonly observed chromosomal gain in our cohort, as a marker of chromosomal gain. Samples were scored as having arm gain or loss if more than 50% of the arm was gained or lost according to the empirically determined cutoffs.

Subgroup identification and validation

Consensus bootstrapped non-negative matrix factorisation (NMF) clustering was performed on the 10,000 most variably methylated probes (identified by measuring the standard deviation of M scores[5]), as previously described[1]. Briefly, we employed NMF to identify from 3 to 10 metagenes from repeated sampling (n=250) of

80% of the dataset, followed by metagene projection onto the entire dataset), which were then clustered using k-means into 3 to 10 clusters.

Metagenes are a single score that reflects the methylation status of multiple CpG loci, and can be thought of as being representative of the major biological effects within the dataset. Metagenes as derived by NMF provide a quantitative measurement of multiple distinct "real-world" (i.e. non-abstract) biological signatures, which in this instance primarily describe the major differences amongst the described sub-groups. A single score represents the weighted contribution of multiple CpGs, but it does not follow that all CpGs must contribute equally to a metagene nor that that each contribution must be hypo- or hyper methylated; directionality is not implied but may be elucidated post-hoc.

Cluster stability measures (Cohen's kappa, average silhouette scores) were assessed to identify optimal combinations of metagenes and resultant clusters. Samples assigned to the same cluster in fewer than 80% of replicates were classified as not classifiable (NC). After assignment of sample cluster, subgroups were visualised by applying *t*-Distributed Stochastic Neighbour Embedding (t-SNE) dimensionality reduction to identify three components, using the R package Rtsne (https://cran.r-project.org/web/packages/Rtsne/index.html; date accessed Mar 2nd, 2017). The quality of the identified cluster subgroups was further assessed using silhouette plots[6]. In order to validate the observed metagene patterns and identified subgroups, we projected metagenes[7] from a non-overlapping cohort of 276 medulloblastomas (GSE54880[8]) run on the 450k array, and classified them into the 7 primary subgroups using an optimised support vector machine (SVM) learning classifier that was trained on the 428 medulloblastoma primary cohort.

Subgroup characterisation

Histopathological disease subtypes were assessed by central review from three experienced neuro-pathologists, according to current WHO criteria[9]. Where central review was unavailable, local pathological diagnoses were used. Chang's criteria were used to assign metastatic stage[10]. Since imaging was not available in many cases, M- disease was defined as M0, M1 or M0/1 disease, and M+ disease as M2+. Tumours were classed as R+ if their residuum following excision exceeded 1.5cm^2.

Amplification of the *MYC* and *MYCN* oncogenes was assessed by iFISH (interphase fluorescent *in situ* hybridsation), and, where not available, multiplex ligation dependent probe amplification (MLPA) and/or estimation of copy number from methylation array locus intensities. Mutations in exons 4 to 9 of *TP53* and in the *TERT* promoter were assessed as previously[11,12].

Subgroup-specific differentially methylated CpG loci were identified using the R package limma[13]. In order to identify differentially methylated regions (DMRs), gene-proximal DMRs, defined as being within 10kb of a gene, were identified using the R package DMRcate[14]. In order to derive a single measure of methylation from these DMRs, the maximally changed region was identified by initially identifying the most differential CpG locus within each DMR, then expanding the region to include at least two CpG loci, whilst maximising the average methylation difference, continuing to expand the maximally changed region until the average change in methylation started to decrease. The reported methylation for each DMR was therefore the average of the maximally changed region. In order to investigate the characteristics of differentially methylated genes, the R package missMethyl[15] was used to identify KEGG pathway enrichments from lists of subgroup-specific hypo-

methylated loci, with a methylation beta difference > 0·3, whilst correcting for the potential bias conferred by having different numbers of probes mapping to different genes. Significant pathway enrichments are summarised in supplementary Table 2. Subgroup-specific age-differences between the newly-characterised nonMB$_{WNT}$/nonMB$_{SHH}$ medulloblastoma subgroups were identified using ANOVA.

Transcriptome analysis

Transcriptome sequencing was available for 190/428 primary medulloblastomas in the methylation array cohort. cDNA libraries were prepared using TruSeq RNA Library Kit and 100bp paired-end sequencing was performed on an Illumina HiSeq2500 to produce ~90M reads per sample. Samples were quality controlled using Fastqc, aligned to hg19 using RNA-star[16] and gene expression quantified by counting reads mapped to the Gencodev17 library using HT-Seq (Union mode)[17]. Variance stabilising transformation, normalisation to overall library size, and differential expression were performed using DESeq2[18]. GOseq (Wallenius approximation option)[19] was used for pathway enrichment analysis on the differentially expressed gene set, whilst correcting for selection bias, *i.e.* genes with a higher number of reads (more highly expressed or a longer transcript) are more likely to be detected as differentially expressed.

Mutational analysis

To subgroup additional MB$_{SHH}$ cases from an external dataset for which genome-sequencing data was available[20], the likelihood of belonging to either MB$_{SHH\text{-}Child}$ or MB$_{SHH\text{-}Infant}$, given the age at diagnosis, was assessed by modelling the age distribution of the MB$_{SHH}$ subgroups in our cohort and applying it to the external dataset. Samples from the external dataset were assigned to either subgroup if their odds-ratio of membership exceeded 3:1.

Genomic copy number specific analysis

Cytogenetic changes were assessed for their association with subgroup using chi-squared tests. Changes were reported if present in at least 10% of one or more subgroups and with a subgroup enrichment with adjusted $p < 0{\cdot}05$.

Survival analysis

We performed Cox modelling only on patients who have received comparable treatment, i.e. limiting cohorts to patients aged from 3-16 years, who received maximal surgical resection and cranio-spinal irradiation with curative intent, these being the most important factors affecting prognosis. Whilst there has been some heterogeneity of treatment, all received cranio-spinal radiotherapy with a dose that was appropriate to their clinical risk factors (i.e. M+ disease, LCA) and the chemotherapy that was used was standard for the day (e.g. SJMB, Milan, POG 9031 for high risk) or for standard risk disease (e.g. CCNU, Cisplatin and Vincristine (Packer) or PNET 3).

Established risk factors for the MB$_{SHH\text{-}Child}$ subgroup were assessed in an expanded MB$_{SHH}$ cohort (<16 years old at diagnosis with cranio-spinal irradiation and maximal surgical resection), augmenting samples classed as MB$_{SHH\text{-}Child}$ by methylation array (n=24) with MB$_{SHH}$ samples for which confirmatory 450k methylation microarray-based subgrouping was unavailable. As before, MB$_{SHH}$ samples were added to this cohort if their odds ratio of membership of the MB$_{SHH\text{-}Child}$ subgroup based on their age at diagnosis exceeded 3:1 (n=31). Since the expanded MB$_{SHH\text{-}Child}$ cohort included samples for which cytogenetic changes were unavailable, we tested currently understood SHH-specific risk markers (*MYCN* amplification, *TP53* mutation, *TERT* mutation) and

clinical parameters (metastatic stage, residual disease, LCA pathology, gender) in univariate and multivariate Cox models in this cohort. All potential variables were tested for proportionality of hazards in univariate and multivariate models.

For the non-MB_{SHH}/non-MB_{WNT} medulloblastoma subgroups, the newly discovered high-risk metagene and genomic copy-number changes were assessed in univariate and cross-validated Cox proportional hazard survival models on childhood cohorts (aged 3·0-16·0 years) that had uniformly received cranio-spinal irradiation. Genomic copy-number changes were filtered before being considered as prognostic markers: Putative survival markers had to be (i) observed in at least 10% of samples within the subgroup of interest, and (ii) be significant (unadjusted $p<0.05$) in log-rank tests of survival. Subsequently, all potential variables were tested for proportionality of hazards in univariate and multivariate models. *MYC* amplification showed a non-proportionality of hazards due to its differential, subgroup-specific disease outcomes that disappeared when MB_{Grp3} and MB_{Grp4} medulloblastomas were considered separately (*MYC* amplified MB_{Grp4} did not have the dismal outcomes observed in *MYC* amplified MB_{Grp3} tumours). In addition to the cytogenetic factors defined above, the prognostic potential of established disease-wide features (metastatic disease, LCA pathology, residual disease, gender, *MYC*/*MYCN* amplification, i17q), as well as other molecular features (membership of high-risk methylomic group, defined by metagene V1, membership of MB_{Grp3}) was tested where appropriate.

For all multivariate survival modelling, missing data were classified as 'missing at random' and samples with missing data were therefore removed from the analysis. Subsequently, prognostic markers were identified in semi-supervised multivariate survival models by performing 100 rounds of 10 fold-cross-validation. In each round of cross-validation, the predictive performance of each factor was assessed by calculating area under the curve (AUC) from survival-dependent Receiver-Operating Characteristic (ROC) curves at 5 years in the left-out fold using the R package risksetROC (https://cran.r-project.org/web/packages/risksetROC/index.html; date accessed Mar 2nd, 2017), and the mean AUC from the 10 folds was subsequently calculated. A final mean AUC was calculated from the 100 repetitions. Markers were entered into the model if a cross-validated increase in AUC was observed. Limits for the total number of included markers were dependent upon the sample size of the discovery cohort. Cox proportional hazards models were constructed for the identified risk markers in each cohort and reported in Tables 2,3 and Supplementary Figure 7.

To identify clinically relevant risk stratification schemes, the cross-validated, multivariate Cox models were used to predict survival at 5 years; patients were stratified according to their predicted survival, dividing predicted outcomes into two or three risk groups as appropriate, and their constituent risk factor profiles were used to derive pragmatic schemes for assigning patient risk (Supplementary Figure 4B, Supplementary Figure 6, Supplementary Figure 7B,F).

The performance of the proposed risk stratification schemes was assessed by measuring AUC at 5 years and comparing against the SIOP-PNET5 non-MB_{WNT} stratification scheme (high risk: Positive for any combination of LCA pathology, metastatic disease, residual disease and *MYC(N)* amplification; standard risk: Negative for the above markers) and a recently described subgroup-directed cytogenetic stratification scheme[21].

All bioinformatic and statistical analyses were performed using R (v3·2·3). Risk stratification was performed using progression-free survival (PFS) times, defined as time elapsed from diagnosis to first event, which was tumour recurrence or progression.

Supplementary Methods References

1 Schwalbe EC, Williamson D, Lindsey JC, *et al.* DNA methylation profiling of medulloblastoma allows robust subclassification and improved outcome prediction using formalin-fixed biopsies. *Acta Neuropathol* 2013; **125**: 359–71.

2 Aryee MJ, Jaffe AE, Corrada-Bravo H, *et al.* Minfi: a flexible and comprehensive Bioconductor package for the analysis of Infinium DNA methylation microarrays. *Bioinformatics* 2014; **30**: 1363–9.

3 Touleimat N, Tost J. Complete pipeline for Infinium(®) Human Methylation 450K BeadChip data processing using subset quantile normalization for accurate DNA methylation estimation. *Epigenomics* 2012; **4**: 325–41.

4 Nordlund J, Bäcklin CL, Wahlberg P, *et al.* Genome-wide signatures of differential DNA methylation in pediatric acute lymphoblastic leukemia. *Genome Biol* 2013; **14**: r105.

5 Du P, Zhang X, Huang C-C, *et al.* Comparison of Beta-value and M-value methods for quantifying methylation levels by microarray analysis. *BMC Bioinformatics* 2010; **11**: 587.

6 Rousseeuw PJ. Silhouettes: A graphical aid to the interpretation and validation of cluster analysis. *Journal of Computational and Applied Mathematics* 1987; **20**: 53–65.

7 Tamayo P, Scanfeld D, Ebert BL, Gillette MA, Roberts CWM, Mesirov JP. Metagene projection for cross-platform, cross-species characterization of global transcriptional states. *Proc Natl Acad Sci USA* 2007; **104**: 5959–64.

8 Hovestadt V, Remke M, Kool M, *et al.* Robust molecular subgrouping and copy-number profiling of medulloblastoma from small amounts of archival tumour material using high-density DNA methylation arrays. *Acta Neuropathol* 2013; **125**: 913–6.

9 Louis DN, Perry A, Reifenberger G, *et al.* The 2016 World Health Organization Classification of Tumors of the Central Nervous System: a summary. *Acta Neuropathol* 2016; **131**: 803–20.

10 Chang CH, Housepian EM, Herbert C. An operative staging system and a megavoltage radiotherapeutic technic for cerebellar medulloblastomas. *Radiology* 1969; **93**: 1351–9.

11 Lindsey JC, Schwalbe EC, Potluri S, Bailey S, Williamson D, Clifford SC. TERT promoter mutation and aberrant hypermethylation are associated with elevated expression in medulloblastoma and characterise the majority of non-infant SHH subgroup tumours. *Acta Neuropathol* 2014; **127**: 307–9.

12 Lindsey JC, Hill RM, Megahed H, *et al.* TP53 mutations in favorable-risk Wnt/Wingless-subtype medulloblastomas. *J Clin Oncol* 2011; **29**: e344–6–authorreplye347–8.

13 Ritchie ME, Phipson B, Wu D, *et al.* limma powers differential expression analyses for RNA-sequencing and microarray studies. *Nucleic Acids Res* 2015; **43**: e47–7.

14 Peters TJ, Buckley MJ, Statham AL, *et al.* De novo identification of differentially methylated regions in the human genome. *Epigenetics Chromatin* 2015; **8**: 6.

15 Phipson B, Maksimovic J, Oshlack A. missMethyl: an R package for analyzing data from Illumina's HumanMethylation450 platform. *Bioinformatics* 2016; **32**: 286–8.

16 Dobin A, Davis CA, Schlesinger F, *et al.* STAR: ultrafast universal RNA-seq aligner. *Bioinformatics* 2013; **29**: 15–21.

17 Anders S, Pyl PT, Huber W. HTSeq--a Python framework to work with high-throughput sequencing data. *Bioinformatics* 2015; **31**: 166–9.

18 Love MI, Huber W, Anders S. Moderated estimation of fold change and dispersion for RNA-seq data with DESeq2. *Genome Biol* 2014; **15**: 550.

19 Young MD, Wakefield MJ, Smyth GK, Oshlack A. Gene ontology analysis for RNA-seq: accounting for selection bias. *Genome Biol* 2010; **11**: R14.

20 Kool M, Jones DTW, Jäger N, *et al.* Genome sequencing of SHH medulloblastoma predicts genotype-related response to smoothened inhibition. *Cancer Cell* 2014; **25**: 393–405.

21 Shih DJH, Northcott PA, Remke M, *et al.* Cytogenetic prognostication within medulloblastoma subgroups. *J Clin Oncol* 2014; **32**: 886–96.

Supplementary Figures and Tables

A

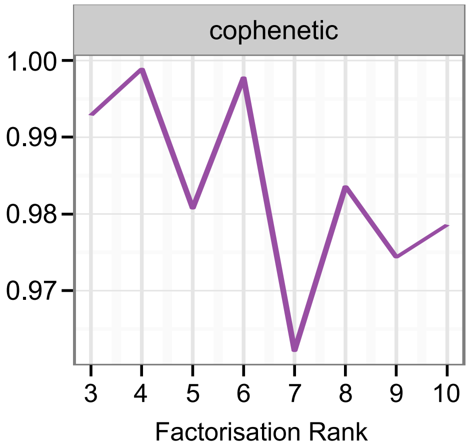

B

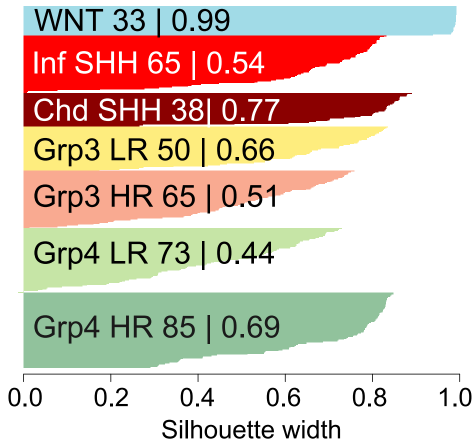

C

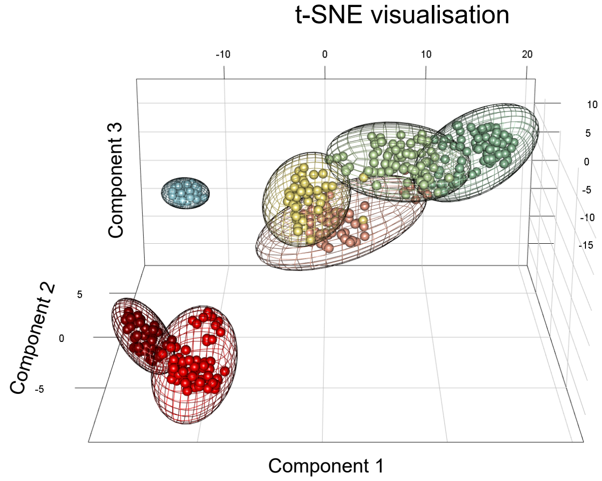

D

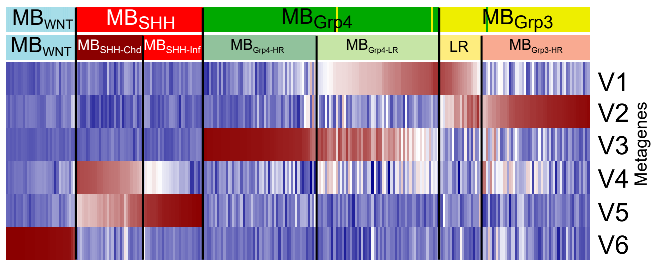

E

7 group classification - this study

	Original classification - Hovestadt *et al.* call			
	WNT	SHH	Grp3	Grp4
WNT	33	0	0	0
SHH-Infant	0	28	0	0
SHH-Child	0	32	0	0
Grp3-LR	0	0	20	0
Grp3-HR	0	0	50	1
Grp4-LR	0	0	2	56
Grp4-HR	0	0	0	54

F

428 MB group classification

	704 MB group classification						
	WNT	SHH-Infant	SHH-Child	Grp3-LR	Grp3-HR	Grp4-LR	Grp4-HR
WNT	33	0	0	0	0	0	0
SHH-Infant	0	65	0	0	0	0	0
SHH-Child	0	0	38	0	0	0	0
Grp3-LR	0	0	0	50	0	0	0
Grp3-HR	0	0	0	0	65	0	0
Grp4-LR	0	0	0	0	0	73	0
Grp4-HR	0	0	0	0	0	0	85

G

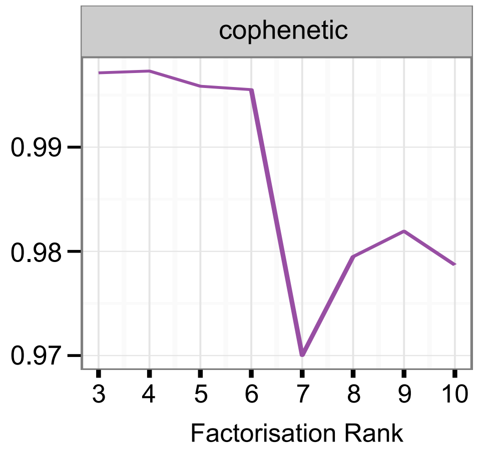

H

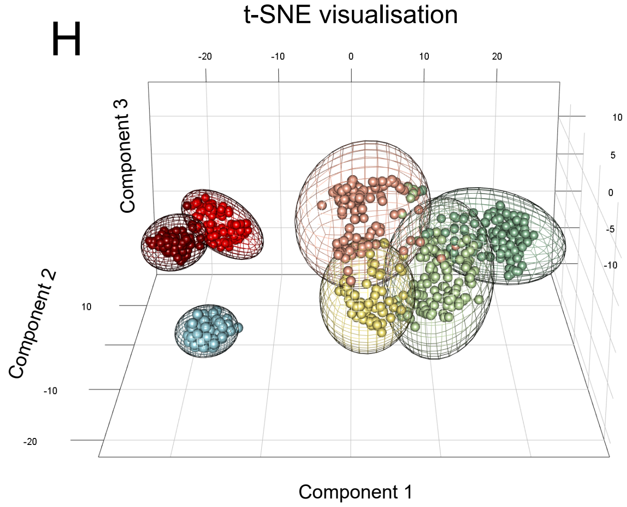

Supplementary Figure 1. Medulloblastoma comprises 7 reproducible, methylation-dependent subgroups. A. Cophenetic coefficient plot for factorisation ranks from 3 to 10 from consensus NMF clustering identifies 6 metagenes as optimal. **B.** Silhouette plot demonstrates robust subgroup assignment. The number of members and average silhouette width of each subgroup is shown. **C.** t-SNE visualisation of 10,000 most variably methylated loci. Tumours are plotted as spheres, coloured by their NMF-based subgroup assignment (blue – MB_{WNT}; dark red – $MB_{SHH\text{-}Child}$; red $MB_{SHH\text{-}Infant}$; darker green – $MB_{Grp4\text{-}HR}$; green – $MB_{Grp4\text{-}LR}$; peach – $MB_{Grp3\text{-}HR}$; yellow – $MB_{Grp3\text{-}LR}$. Covariance spheroids were plotted across 95% confidence intervals. **D.** Validation of novel subgroups (second row)[8] in an independent cohort of 276 medulloblastomas. NMF metagene projection recapitulated previously identified medulloblastoma subgroups, which were congruent with their original subgroup assignment (first row)[8]. **E.** Validation of identified subgroups in an independent medulloblastoma 450k DNA methylation array dataset. Confusion matrix shows original subgroup calls from Hovestadt *et al.*'s dataset (GSE54880) in columns[8]. Subgroup calls assigned by an SVM classifier are shown in rows for the 7 subgroups identified in this study. **F.** Subgroup assignments remain stable when clustering with discovery (n=428) or combined discovery and validation cohorts (n=704). Table shows consensus subgroup assignments in discovery cohort compared with subgroup assignments of discovery cohort non-NC tumours (n=409) when clustered as part of the combined cohort. **G.** Subgroup discovery in the combined discovery and validation cohort (n=704) does not identify additional subgroup heterogeneity. Cophenetic coefficients are shown for factorisation ranks from 3 to 10. **H.** t-SNE visualisation of 10,000 most variably methylated loci for the combined cohort. Tumours are plotted as spheres and coloured by NMF-based subgroup assignment (blue – MB_{WNT}; dark red – $MB_{SHH\text{-}Child}$; red – $MB_{SHH\text{-}Infant}$; darker green – $MB_{Grp4\text{-}HR}$; green – $MB_{Grp4\text{-}LR}$; peach – $MB_{Grp3\text{-}HR}$; yellow – $MB_{Grp3\text{-}LR}$). Covariance spheroids were plotted across 95% confidence intervals.

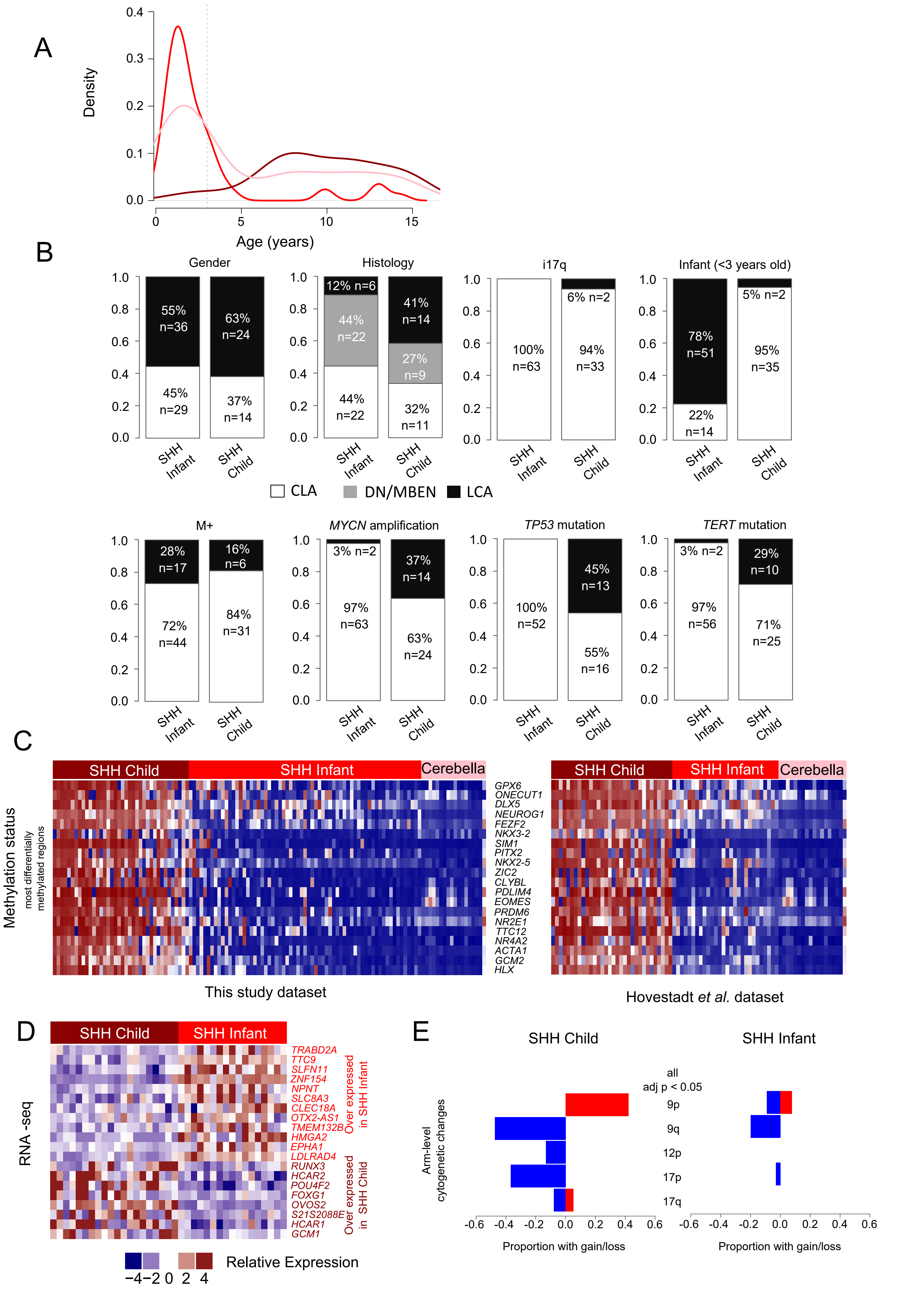
A
Density
Age (years)
B
Gender
Histology
i17q
Infant (<3 years old)
SHH Infant
SHH Child
CLA
DN/MBEN
LCA
M+
MYCN amplification
TP53 mutation
TERT mutation
C
SHH Child
SHH Infant
Cerebella
Methylation status
most differentially methylated regions
GPX6
ONECUT1
DLX5
NEUROG1
FEZF2
NKX3-2
SIM1
PITX2
NKX2-5
ZIC2
CLYBL
PDLIM4
EOMES
PRDM6
NR2E1
TTC12
NR4A2
ACTA1
GCM2
HLX
This study dataset
Hovestadt et al. dataset
D
RNA -seq
TRABD2A
TTC9
SLFN11
ZNF154
NPNT
SLC8A3
CLEC18A
OTX2-AS1
TMEM132B
HMGA2
EPHA1
LDLRAD4
RUNX3
HCAR2
POU4F2
FOXG1
OVOS2
S21S2088E
HCAR1
GCM1
Over expressed in SHH Infant
Over expressed in SHH Child
Relative Expression
E
SHH Child
SHH Infant
all adj p < 0.05
9p
9q
12p
17p
17q
Arm-level cytogenetic changes
Proportion with gain/loss

Supplementary Figure 2. Characterisation and molecular validation of identified $MB_{SHH\text{-}Infant}$ and $MB_{SHH\text{-}Child}$ subgroups. **A.** Density plots of age distributions are shown for individual $MB_{SHH\text{-}Infant}$ and $MB_{SHH\text{-}Child}$ subgroups (red and dark red respectively), alongside a single MB_{SHH} entity, shown in pink. **B.** Incidences of established clinico-pathological and molecular correlates based on available data are shown as stacked percentage bar plots. **C.** Top 20 gene-proximal differentially methylated regions (DMRs defined by the average methylation of maximally changed gene-proximal regions identified using the R package DMRcate) and methylation of 18 normal cerebella are shown**.** Recapitulation of identified DMR changes in an independent validation cohort[8] is shown on right-hand side**.** **D.** Identification of MB_{SHH} subgroup differentially expressed genes. Heatmap displays Z scores from VSD-transformed RNA-seq data of MB_{SHH} tumours. Genes over-expressed in $MB_{SHH\text{-}Infant}$ disease are labelled red; genes over-expressed in $MB_{SHH\text{-}Child}$ disease are labelled dark red. **E.** MB_{SHH} subgroups show differential cytogenetic correlates. Incidence of arm-level chromosomal gains (red) / losses (blue) significantly different between childhood and infant SHH subgroups (adjusted $p < 0{\cdot}05$).

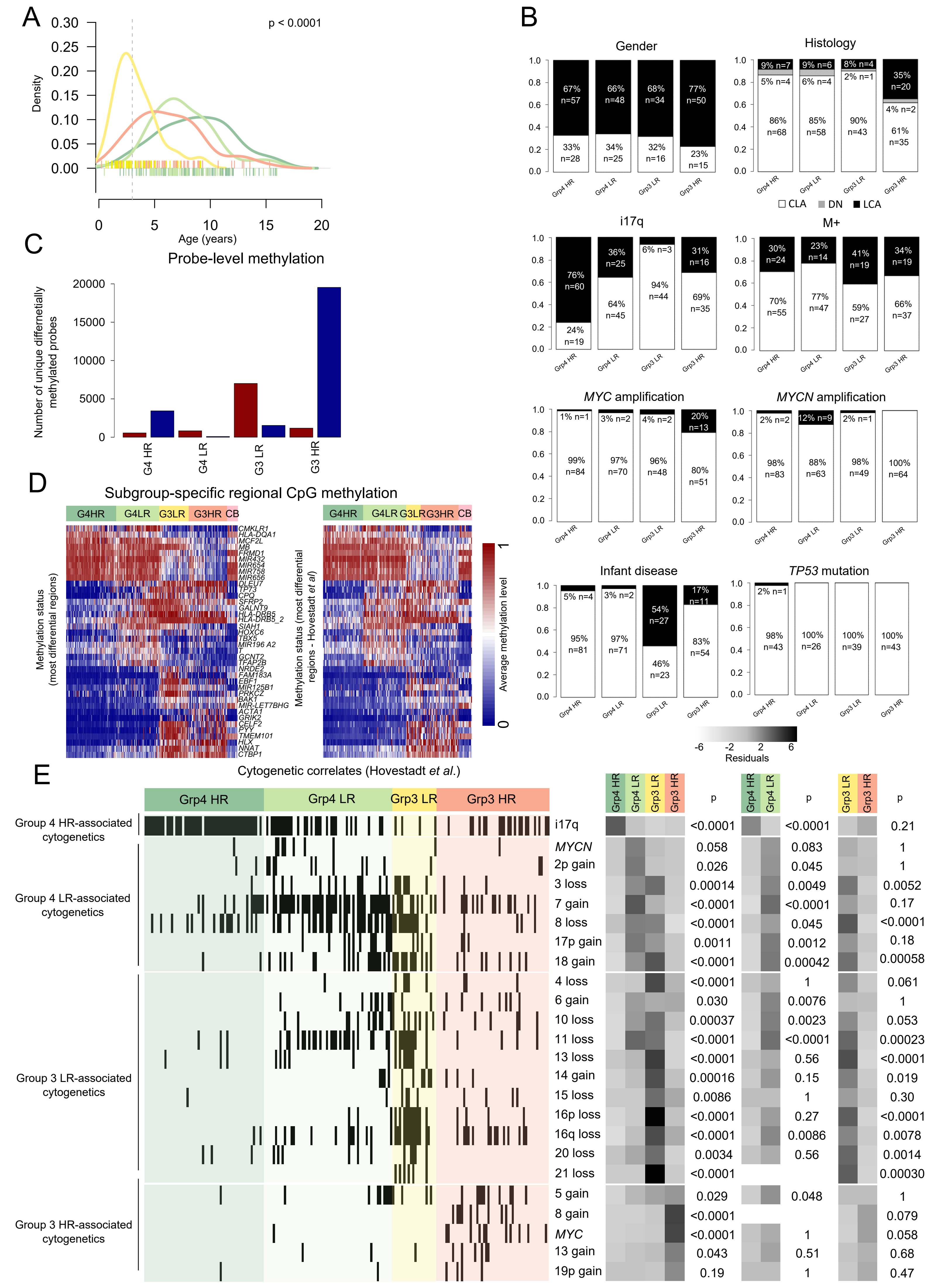
A
p < 0.0001
Density
0.30
0.25
0.20
0.15
0.10
0.05
0.00
0
5
10
15
20
Age (years)
B
Gender
Grp4 HR 67% n=57, 33% n=28
Grp4 LR 66% n=48, 34% n=25
Grp3 LR 68% n=34, 32% n=16
Grp3 HR 77% n=50, 23% n=15
Histology
Grp4 HR 9% n=7, 5% n=4, 86% n=68
Grp4 LR 9% n=6, 6% n=4, 85% n=58
Grp3 LR 8% n=4, 2% n=1, 90% n=43
Grp3 HR 35% n=20, 4% n=2, 61% n=35
CLA
DN
LCA
i17q
Grp4 HR 76% n=60, 24% n=19
Grp4 LR 36% n=25, 64% n=45
Grp3 LR 6% n=3, 94% n=44
Grp3 HR 31% n=16, 69% n=35
M+
Grp4 HR 30% n=24, 70% n=55
Grp4 LR 23% n=14, 77% n=47
Grp3 LR 41% n=19, 59% n=27
Grp3 HR 34% n=19, 66% n=37
MYC amplification
Grp4 HR 1% n=1, 99% n=84
Grp4 LR 3% n=2, 97% n=70
Grp3 LR 4% n=2, 96% n=48
Grp3 HR 20% n=13, 80% n=51
MYCN amplification
Grp4 HR 2% n=2, 98% n=83
Grp4 LR 12% n=9, 88% n=63
Grp3 LR 2% n=1, 98% n=49
Grp3 HR 100% n=64
Infant disease
Grp4 HR 5% n=4, 95% n=81
Grp4 LR 3% n=2, 97% n=71
Grp3 LR 54% n=27, 46% n=23
Grp3 HR 17% n=11, 83% n=54
TP53 mutation
Grp4 HR 2% n=1, 98% n=43
Grp4 LR 100% n=26
Grp3 LR 100% n=39
Grp3 HR 100% n=43
-6
-2
2
6
Residuals
C
Probe-level methylation
Number of unique differentially methylated probes
20000
15000
10000
5000
0
G4 HR
G4 LR
G3 LR
G3 HR
D
Subgroup-specific regional CpG methylation
G4HR
G4LR
G3LR
G3HR
CB
Methylation status (most differential regions)
Methylation status (most differential regions - Hovestadt et al)
Average methylation level
1
0
CMKLR1
HLA-DQA1
MCF2L
MB
FRMD1
MIR432
MIR654
MIR758
MIR656
DLEU7
TP73
CPQ
SFRP2
GALNT9
HLA-DRB5
HLA-DRB5_2
SIAH1
HOXC6
TBX5
MIR196 A2
T
GCNT2
TFAP2B
NRDE2
FAM183A
EBF1
MIR125B1
PRKCZ
BAK1
MIR-LET7BHG
ACTA1
GRIK2
CELF2
PYY
TMEM101
HLX
NNAT
CTBP1
E
Cytogenetic correlates (Hovestadt et al.)
Grp4 HR
Grp4 LR
Grp3 LR
Grp3 HR
Group 4 HR-associated cytogenetics
Group 4 LR-associated cytogenetics
Group 3 LR-associated cytogenetics
Group 3 HR-associated cytogenetics
p
i17q <0.0001 <0.0001 0.21
MYCN 0.058 0.083 1
2p gain 0.026 0.045 1
3 loss 0.00014 0.0049 0.0052
7 gain <0.0001 <0.0001 0.17
8 loss <0.0001 0.045 <0.0001
17p gain 0.0011 0.0012 0.18
18 gain <0.0001 0.00042 0.00058
4 loss <0.0001 1 0.061
6 gain 0.030 0.0076 1
10 loss 0.00037 0.0023 0.053
11 loss <0.0001 <0.0001 0.00023
13 loss <0.0001 0.56 <0.0001
14 gain 0.00016 0.15 0.019
15 loss 0.0086 1 0.30
16p loss <0.0001 0.27 <0.0001
16q loss <0.0001 0.0086 0.0078
20 loss 0.0034 0.56 0.0014
21 loss <0.0001 0.00030
5 gain 0.029 0.048 1
8 gain <0.0001 0.079
MYC <0.0001 1 0.058
13 gain 0.043 0.51 0.68
19p gain 0.19 1 0.47

Supplementary Figure 3. Characterisation and molecular validation of non-MB$_{SHH}$/non-MB$_{WNT}$ subgroups. **A.** MB$_{Grp3\text{-}LR}$ and MB$_{Grp3\text{-}HR}$ (yellow and peach respectively) subgroups show earlier age of diagnosis than MB$_{Grp4\text{-}LR}$ and MB$_{Grp4\text{-}HR}$ subgroups (green and darker green respectively). Individual patient ages at diagnosis are shown as ticks along the X axis. **A.** Incidences of clinico-pathological and molecular correlates based on available data are shown as stacked percentage bar plots. **C.** MB$_{Grp3\text{-}HR}$ tumours are enriched for hypo-methylation events compared to other subgroups. Bar plot shows the number of subgroup-specific hyper- (red bars) and hypo-methylation (blue bars) events across the four non-MB$_{SHH}$/non-MB$_{WNT}$ medulloblastoma subgroups. **D.** Top 10 gene-proximal differentially methylated regions (DMRs) for each non-MB$_{SHH}$/non-MB$_{WNT}$ subgroup and methylation of 18 normal cerebella are shown (only 8 DMRs reached significance and magnitude of methylation change cutoffs for MB$_{Grp4\text{-}LR}$ comparison) and their recapitulation of identified non-MB$_{SHH}$/non-MB$_{WNT}$ subgroup specific DMR changes in an independent validation cohort[8], shown on right hand side. **E.** Significant cytogenetic changes observed in the discovery cohort and their incidence in validation cohort. Residuals from χ^2 tests indicate where subgroup-enrichment has occurred (dark greys indicate strong relationships), comparing across all subgroups, then within MB$_{Grp3}$ and MB$_{Grp4}$ individually. Scale bar for residuals is shown.

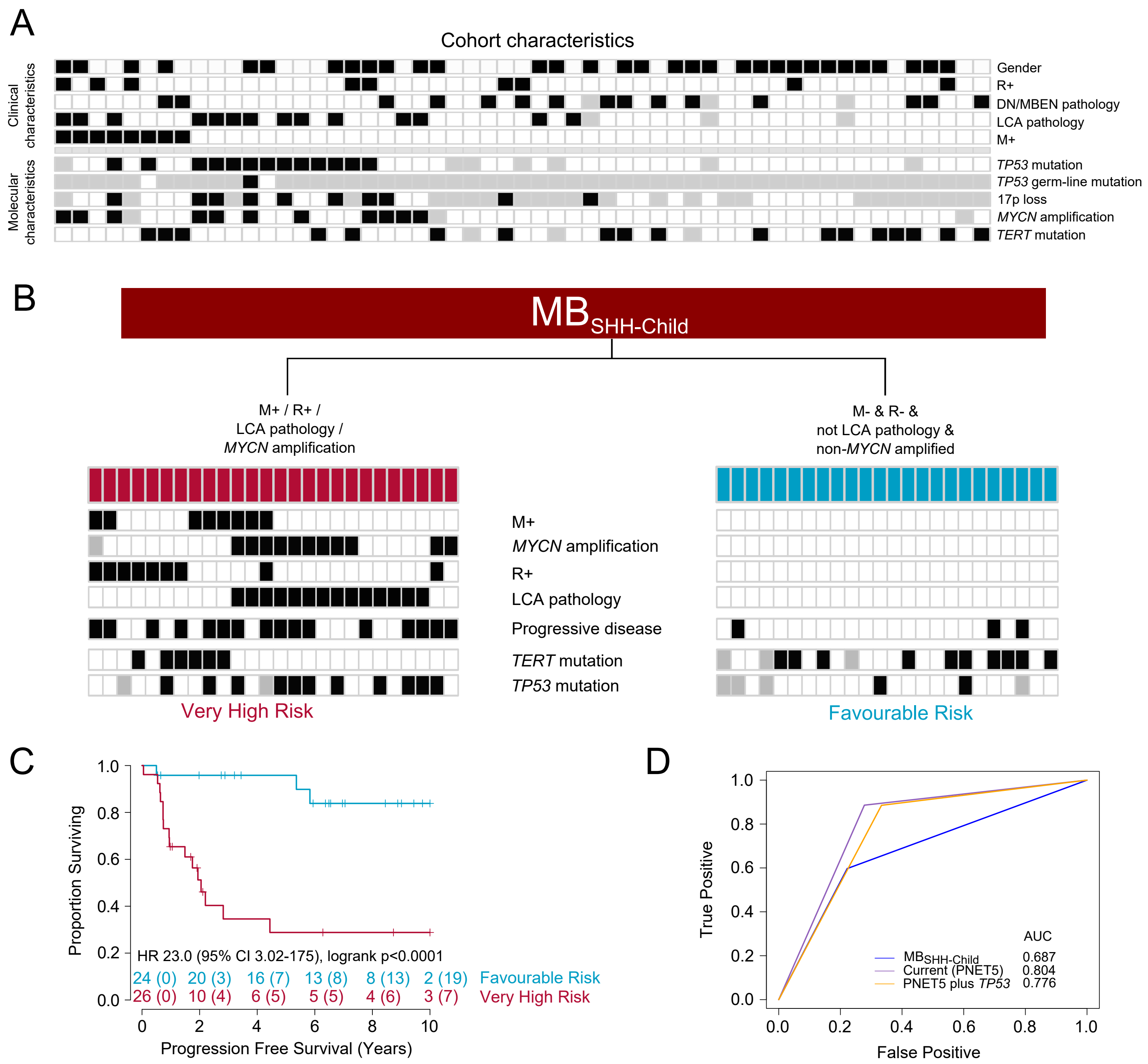
A
Cohort characteristics
Clinical characteristics
Molecular characteristics
Gender
R+
DN/MBEN pathology
LCA pathology
M+
TP53 mutation
TP53 germ-line mutation
17p loss
MYCN amplification
TERT mutation
B
MBSHH-Child
M+ / R+ / LCA pathology / MYCN amplification
M- & R- & not LCA pathology & non-MYCN amplified
M+
MYCN amplification
R+
LCA pathology
Progressive disease
TERT mutation
TP53 mutation
Very High Risk
Favourable Risk
C
Proportion Surviving
HR 23.0 (95% CI 3.02-175), logrank p<0.0001
24 (0) 20 (3) 16 (7) 13 (8) 8 (13) 2 (19) Favourable Risk
26 (0) 10 (4) 6 (5) 5 (5) 4 (6) 3 (7) Very High Risk
Progression Free Survival (Years)
D
True Positive
AUC
MBSHH-Child 0.687
Current (PNET5) 0.804
PNET5 plus TP53 0.776
False Positive

Supplementary Figure 4. Risk stratification of $MB_{SHH\text{-}Child}$ medulloblastoma. A. Age-dependency of MB_{SHH} subgroups enabled expansion of CSI-irradiated $MB_{SHH\text{-}Child}$ cohort for survival analysis (total n=55). Cohort characteristics are shown. Missing data are shown grey. Abbreviations used: R+, residual disease; DN/MBEN, desmoplastic/nodular, medulloblastoma with extensive nodularity; LCA, large-cell/anaplastic; M+, metastatic disease. **B.** The non-MB_{WNT} SIOP-PNET5-MB survival model for risk stratification of $MB_{SHH\text{-}Child}$ (positive for one or more of LCA pathology / M+ disease / R+ disease / *MYC(N)* amplification are high risk, absent for all high risk features, standard risk), in addition to *TERT* and *TP53* mutation status, shown for information. Positivity for a marker is shown black; missing data are shown grey. **C.** Progression-free survival plots for identified risk subgroups shown in **C**. At risk table (number censored in parentheses) is shown. **D.** Time-dependent ROC curves at 5 years are shown for non-MB_{WNT} SIOP-PNET5-MB clinical trial stratification, in addition to non-MB_{WNT} SIOP-PNET5-MB stratification, plus *TP53* mutation as a poor risk factor, and the empirically determined scheme shown in Table 2 (positive for one or more of *MYCN* amplification, M+ disease or *TP53* mutation).

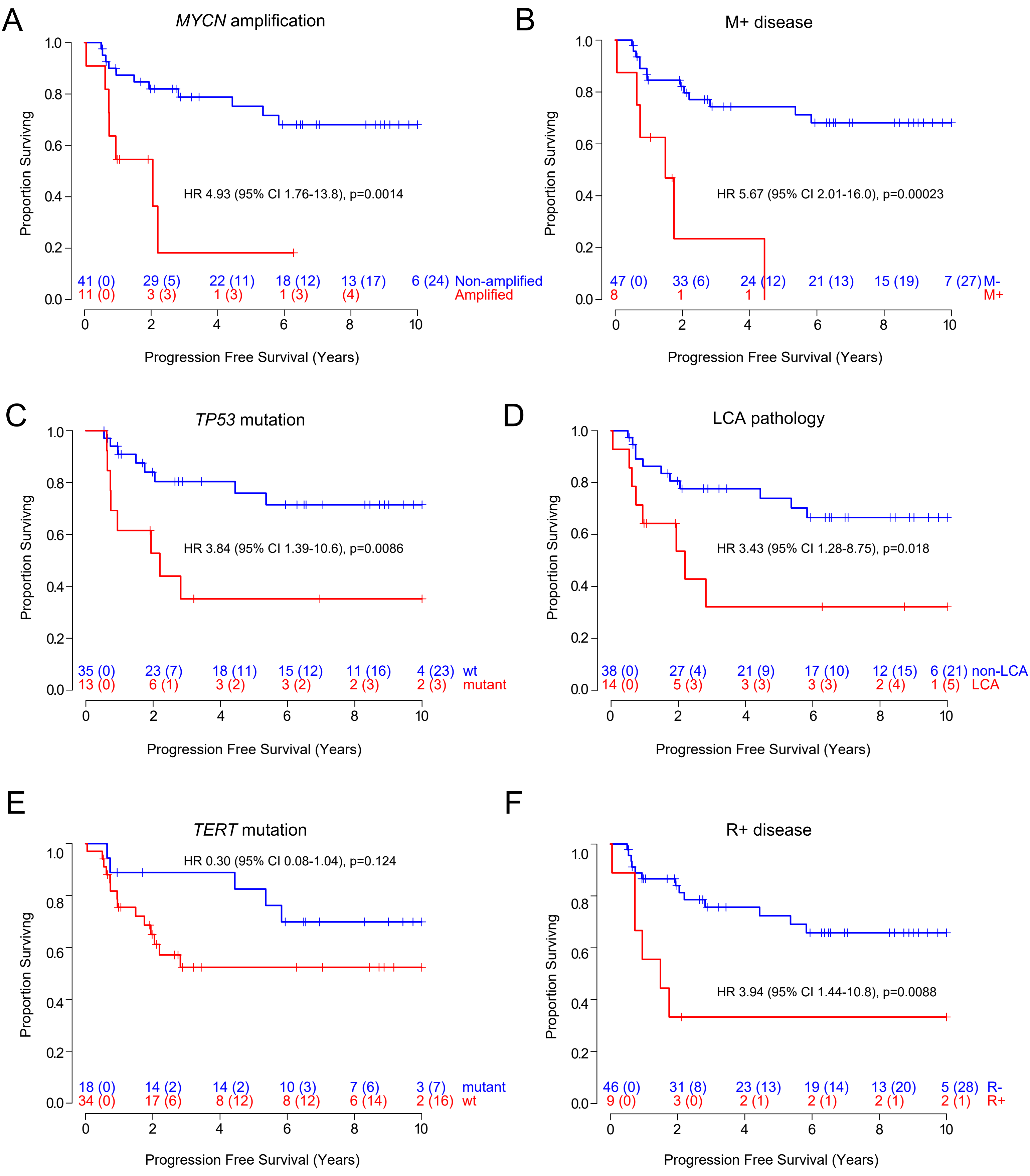

A
MYCN amplification
HR 4.93 (95% CI 1.76-13.8), p=0.0014
41 (0) 29 (5) 22 (11) 18 (12) 13 (17) 6 (24) Non-amplified
11 (0) 3 (3) 1 (3) 1 (3) (4) Amplified
Proportion Survivng
Progression Free Survival (Years)
B
M+ disease
HR 5.67 (95% CI 2.01-16.0), p=0.00023
47 (0) 33 (6) 24 (12) 21 (13) 15 (19) 7 (27) M-
8 1 1 M+
Proportion Survivng
Progression Free Survival (Years)
C
TP53 mutation
HR 3.84 (95% CI 1.39-10.6), p=0.0086
35 (0) 23 (7) 18 (11) 15 (12) 11 (16) 4 (23) wt
13 (0) 6 (1) 3 (2) 3 (2) 2 (3) 2 (3) mutant
Proportion Survivng
Progression Free Survival (Years)
D
LCA pathology
HR 3.43 (95% CI 1.28-8.75), p=0.018
38 (0) 27 (4) 21 (9) 17 (10) 12 (15) 6 (21) non-LCA
14 (0) 5 (3) 3 (3) 3 (3) 2 (4) 1 (5) LCA
Proportion Survivng
Progression Free Survival (Years)
E
TERT mutation
HR 0.30 (95% CI 0.08-1.04), p=0.124
18 (0) 14 (2) 14 (2) 10 (3) 7 (6) 3 (7) mutant
34 (0) 17 (6) 8 (12) 8 (12) 6 (14) 2 (16) wt
Proportion Survivng
Progression Free Survival (Years)
F
R+ disease
HR 3.94 (95% CI 1.44-10.8), p=0.0088
46 (0) 31 (8) 23 (13) 19 (14) 13 (20) 5 (28) R-
9 (0) 3 (0) 2 (1) 2 (1) 2 (1) 2 (1) R+
Proportion Survivng
Progression Free Survival (Years)

Supplementary Figure 5. Investigation of the prognostic significance of disease features in $MB_{SHH\text{-}Child}$ medulloblastoma. A-F. Kaplan-Meier plots, log-rank tests, HR and 95% CIs and at-risk tables (numbers censored in parentheses) are shown for *MYCN* amplification, M+ disease, *TP53* mutation, LCA pathology, *TERT* mutation and R+ disease in expanded, CSI-irradiated $MB_{SHH\text{-}Child}$ cohort (n=55). Abbreviations used: M+, metastatic disease; LCA, large cell/anaplastic; R+, residual disease.

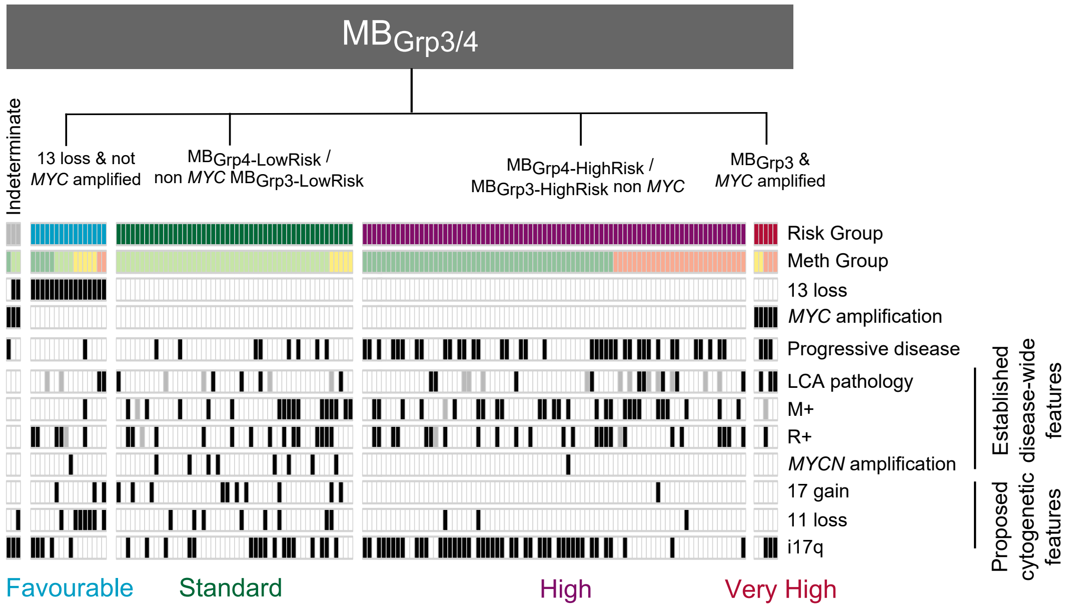
MBGrp3/4
Indeterminate
13 loss & not MYC amplified
MBGrp4-LowRisk / non MYC MBGrp3-LowRisk
MBGrp4-HighRisk / MBGrp3-HighRisk non MYC
MBGrp3 & MYC amplified
Risk Group
Meth Group
13 loss
MYC amplification
Progressive disease
LCA pathology
M+
R+
MYCN amplification
17 gain
11 loss
i17q
Established disease-wide features
Proposed cytogenetic features
Favourable
Standard
High
Very High

Supplementary Figure 6. A novel risk stratification scheme for non-MB_{SHH}/non-MB_{WNT} medulloblastoma. A novel, empirically-derived survival model for risk stratification of childhood MB_{Grp3} and MB_{Grp4} medulloblastoma. Stratification markers are colour coded – Meth group – $MB_{Grp3-HR}$, peach; $MB_{Grp3-LR}$, yellow; $MB_{Grp4-HR}$, darker green; $MB_{Grp4-LR}$, green. 13 loss – presence, black; absence, white. *MYC* - presence, black; absence, white. Established prognostic features identified in disease-wide studies are also shown, in addition to reported cytogenetic prognostic markers (presence, black; absence, white; grey, no data)[21].

A

MB$_{Grp4}$

Covariate	n	HR	95%CI	p	HR	95%CI	p
	Univariate				Cross-validated multivariate (n=97)		
7q gain	112	0.39	0.19-0.82	0.012	0.34	0.16-0.72	0.0054
M+ disease	116	2.63	1.31-5.29	0.0066	2.64	1.31-5.33	0.0068
Male gender	118	2.62	1.09-6.33	0.032			
High risk methylomic group	118	3.11	1.45-6.64	0.0034			
R+ disease	115	1.40	0.70-2.78	0.34			
LCA pathology	108	0.67	0.16-2.78	0.58			
MYCN amplification	117	1.09	0.33-3.57	0.88			
11 loss*	112	0.68	0.21-2.23	0.53			
17 gain*	112	NA	NA	0.031^{+}			

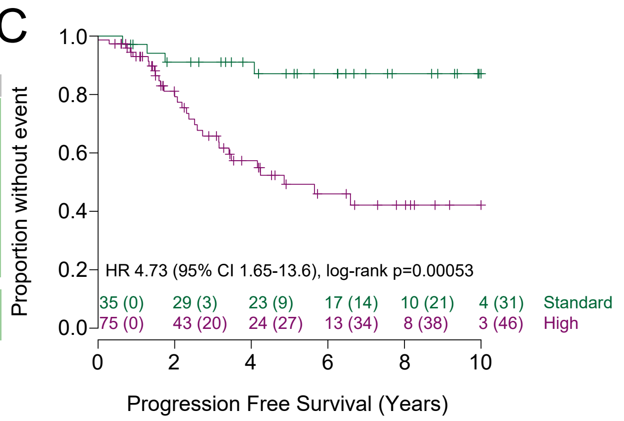

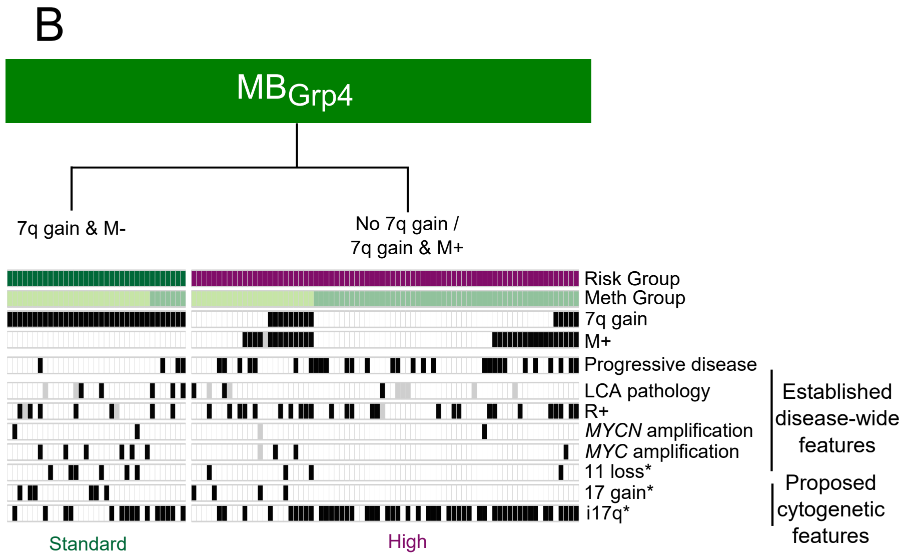

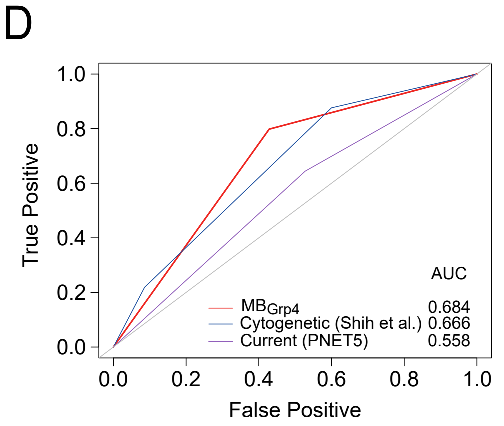

E

MB$_{Grp3}$

Covariate	n	HR	95%CI	p	HR	95%CI	p
	Univariate				Cross-validated multivariate (n=36)		
MYC amplification	56	25.1	3.92-161	0.00067	25.1	3.92-161	0.00067
13q loss	46	0.19	0.03-1.47	0.11			
i17q	46	1.83	0.60-5.58	0.29			
High risk methylomic group	57	4.56	1.07-19.4	0.040			
M+ disease	55	1.11	0.37-2.18	0.82			
LCA pathology	49	1.22	0.45-3.35	0.69			
Male gender	57	0.79	0.34-1.82	0.58			
R+ disease	56	1.14	0.48-2.71	0.77			

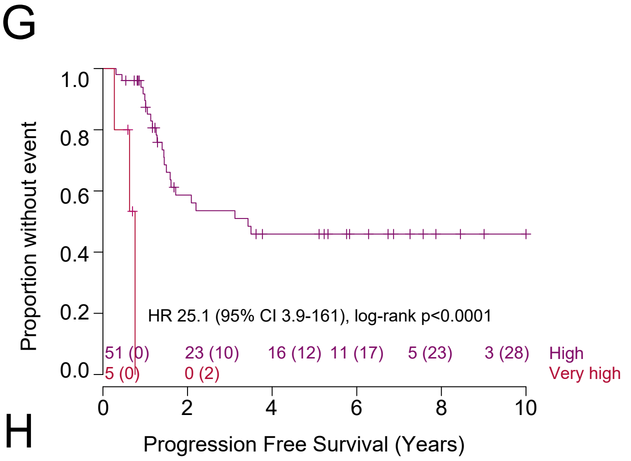

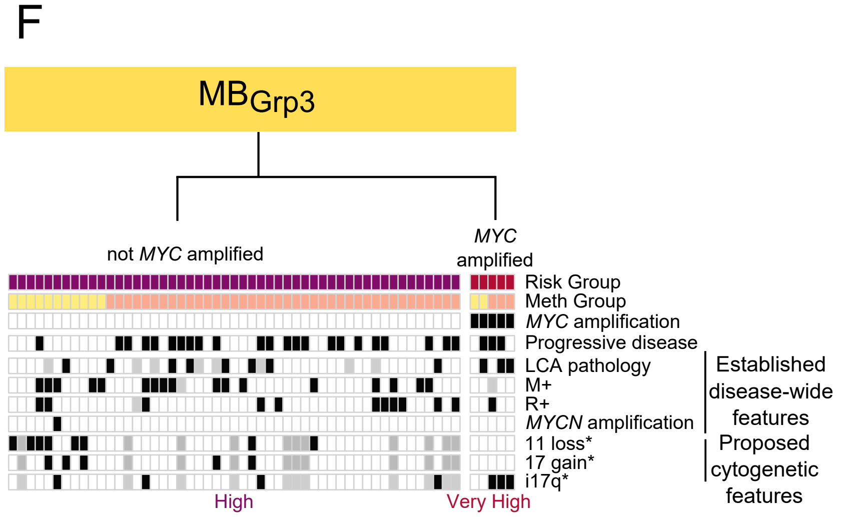

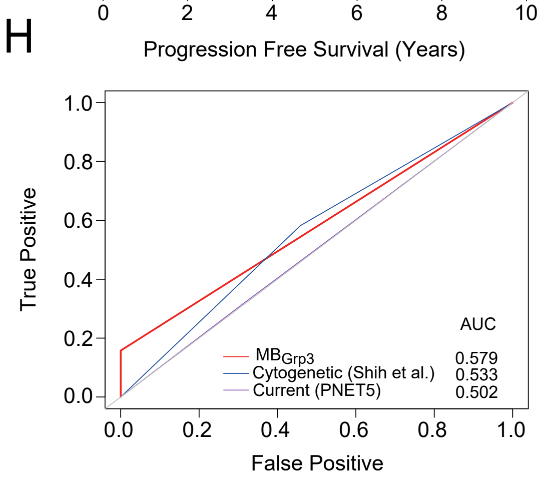

Supplementary Figure 7. Prognostication within MB_{Grp3} or MB_{Grp4} only cohorts. A, E. Identification of prognostic survival markers in combined non-infant MB_{Grp4} (A, n=118) and MB_{Grp3} (E, n=56) medulloblastomas. For each marker, hazard ratio, 95% confidence intervals and p values from Cox Proportional Hazards models are shown. Survival markers that are prognostic in a multivariate survival model are also shown, in addition to reported cytogenetic prognostic markers[21], marked with an asterisk. $^{+}$No events were observed in MB_{Grp4} patients with chromosome 17 gain, so p value from log-rank test is given. **B.** A novel survival model for risk stratification of childhood MB_{Grp4} medulloblastoma (n=113). Stratification markers are colour coded: Risk group – $MB_{Grp4\text{-}HR}$, darker green; $MB_{Grp4\text{-}LR}$, green. 7q gain – presence, black; absence, white. M+ disease – presence, black; absence, white. Established prognostic features identified in disease-wide studies are also shown, in addition to reported cytogenetic prognostic markers[21]. **C.** Progression free survival curves for risk stratified childhood MB_{Grp4} medulloblastoma using scheme outlined in part B. At risk table (numbers censored in parentheses) is shown. **D, H.** Time-dependent ROC curves at 60 months demonstrate performance of novel stratification scheme in MB_{Grp4} (D) and MB_{Grp3} (H) compared to cytogenetic and SIOP-PNET5-MB risk stratifications. **F.** A novel survival model for risk stratification of childhood MB_{Grp3} medulloblastoma (n=56). Stratification markers are colour coded: Meth group – $MB_{Grp3\text{-}HR}$, peach; $MB_{Grp3\text{-}LR}$, yellow. *MYC* amplification – presence, black; absence, white. Established prognostic features identified in disease-wide studies are also shown, in addition to reported cytogenetic prognostic markers[21]. **G.** Progression free survival curves for risk stratified MB_{Grp3} medulloblastoma using scheme outlined in part F. At risk table (numbers censored in parentheses) is shown. Abbreviations used: M+, metastatic disease; LCA, large cell/anaplastic; R+, residual disease.

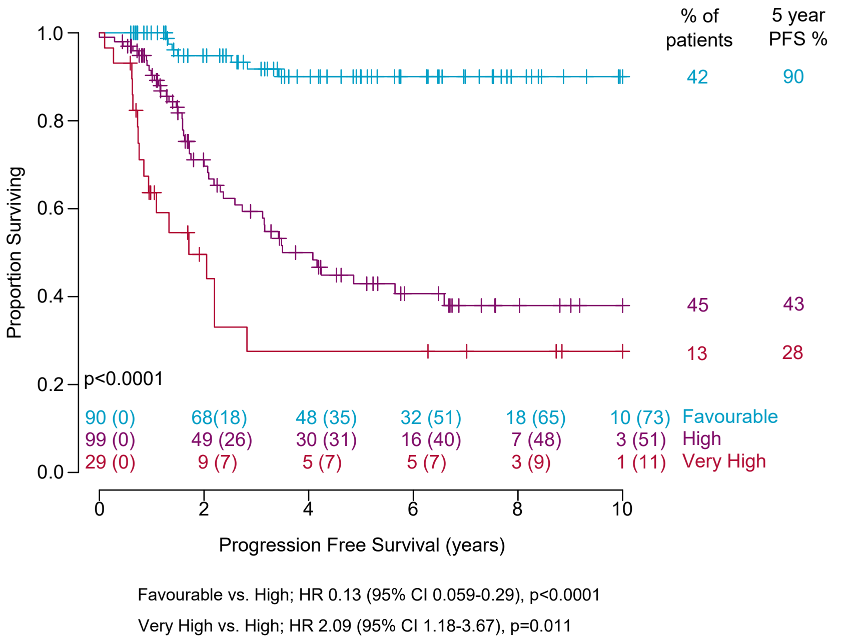

Supplementary Figure 8. Refinement of risk stratification of childhood medulloblastoma, by reclassification of $MB_{Grp4-LR}$ and non-MYC amplified $MB_{Grp3-LR}$ into favorable and high risk groups, by metastatic stage.

Subgroup	Deaths <5 years (%)	Deaths ≥5 years (%)
MB_{WNT}	1 (50%)	1 (50%)
$MB_{SHH\text{-}Child}$	11 (92%)	1 (8%)
$MB_{SHH\text{-}Infant}$	20 (95%)	1 (5%)
$MB_{Grp4\text{-}HighRisk}$	18 (64%)	10 (36%)
$MB_{Grp4\text{-}LowRisk}$	11 (85%)	2 (15%)
$MB_{Grp3\text{-}Lowrisk}$	11 (100%)	0 (0%)
$MB_{Grp3\text{-}HighRisk}$	33 (92%)	3 (8%)

Supplementary Table 1. Characterisation of patterns of late death and progression in novel medulloblastoma subgroups. For each identified subgroup, the number of deaths before and after 5 years is indicated.

Discovery Cohort (Total n = 428)

Demethylated in MB_{WNT} vs normal cerebella

Pathway ID	Pathway	Total n	no. hypo-methylated	P	AdjP
path:hsa04740	Olfactory transduction	357	82	1.3E-49	4.2E-47
path:hsa01100	Metabolic pathways	1178	142	2.5E-43	3.9E-41
path:hsa04080	Neuroactive ligand-receptor interaction	255	43	9.7E-19	1.0E-16
path:hsa04022	cGMP-PKG signaling pathway	162	32	1.3E-14	9.9E-13
path:hsa04261	Adrenergic signaling in cardiomyocytes	144	30	1.9E-14	1.2E-12
path:hsa04151	PI3K-Akt signaling pathway	314	44	4.7E-14	2.4E-12
path:hsa04270	Vascular smooth muscle contraction	117	26	7.1E-14	2.9E-12
path:hsa04020	Calcium signaling pathway	171	32	7.5E-14	2.9E-12
path:hsa04530	Tight junction	133	28	1.0E-13	3.5E-12
path:hsa04014	Ras signaling pathway	219	36	1.2E-13	3.6E-12
path:hsa04024	cAMP signaling pathway	192	33	1.6E-13	4.4E-12
path:hsa04640	Hematopoietic cell lineage	88	20	3.2E-12	8.3E-11
path:hsa04621	NOD-like receptor signaling pathway	150	24	4.3E-11	1.0E-09
path:hsa00230	Purine metabolism	164	26	5.8E-11	1.3E-09
path:hsa04925	Aldosterone synthesis and secretion	80	20	7.5E-11	1.5E-09
path:hsa04514	Cell adhesion molecules (CAMs)	133	24	9.8E-11	1.9E-09
path:hsa05016	Huntington's disease	179	26	1.6E-10	3.0E-09
path:hsa05150	Staphylococcus aureus infection	47	14	2.3E-10	3.9E-09
path:hsa05202	Transcriptional misregulation in cancer	165	27	2.8E-10	4.5E-09
path:hsa04972	Pancreatic secretion	89	19	3.3E-10	5.1E-09

Demethylated in $MB_{SHH\text{-}Child}$ vs $MB_{SHH\text{-}Infant}$

Pathway ID	Pathway	Total n	no. hypo-methylated	P	AdjP
path:hsa04740	Olfactory transduction	357	22	2.7E-19	8.5E-17
path:hsa05206	MicroRNAs in cancer	279	8	1.3E-05	0.0020
path:hsa04014	Ras signaling pathway	219	6	0.0001	0.0124
path:hsa05152	Tuberculosis	156	5	0.0002	0.0124
path:hsa04974	Protein digestion and absorption	78	4	0.0002	0.0136
path:hsa01521	EGFR tyrosine kinase inhibitor resistance	79	4	0.0003	0.0136
path:hsa05146	Amoebiasis	93	4	0.0003	0.0146
path:hsa05200	Pathways in cancer	386	7	0.0004	0.0172
path:hsa04151	PI3K-Akt signaling pathway	314	6	0.0009	0.0305
path:hsa04145	Phagosome	137	4	0.0013	0.0393
path:hsa00770	Pantothenate and CoA biosynthesis	18	2	0.0015	0.0412
path:hsa04512	ECM-receptor interaction	80	3	0.0031	0.0790
path:hsa04658	Th1 and Th2 cell differentiation	88	3	0.0038	0.0905
path:hsa04925	Aldosterone synthesis and secretion	80	3	0.0043	0.0937
path:hsa05310	Asthma	27	2	0.0048	0.0937
path:hsa04211	Longevity regulating pathway	93	3	0.0048	0.0937
path:hsa04320	Dorso-ventral axis formation	28	2	0.0056	0.0984
path:hsa00380	Tryptophan metabolism	37	2	0.0057	0.0984
path:hsa05143	African trypanosomiasis	34	2	0.0062	0.1014
path:hsa04919	Thyroid hormone signaling pathway	114	3	0.0086	0.1286

Demethylated in $MB_{SHH\text{-}Infant}$ vs $MB_{SHH\text{-}Child}$

Pathway ID	Pathway	Total n	no. hypo-methylated	P	AdjP
path:hsa04080	Neuroactive ligand-receptor interaction	255	24	1.6E-18	5.0E-16
path:hsa05202	Transcriptional misregulation in cancer	165	18	6.3E-13	9.8E-11
path:hsa01100	Metabolic pathways	1178	33	5.8E-10	5.1E-08
path:hsa04390	Hippo signaling pathway	150	15	6.6E-10	5.1E-08
path:hsa04724	Glutamatergic synapse	113	13	9.6E-10	6.0E-08
path:hsa05034	Alcoholism	164	13	2.1E-09	1.1E-07
path:hsa04950	Maturity onset diabetes of the young	26	7	6.3E-09	2.8E-07
path:hsa04723	Retrograde endocannabinoid signaling	96	11	1.2E-08	4.5E-07
path:hsa04020	Calcium signaling pathway	171	13	7.2E-08	2.1E-06
path:hsa05322	Systemic lupus erythematosus	113	9	7.4E-08	2.1E-06
path:hsa04550	Signaling pathways regulating pluripotency of stem cells	138	12	7.6E-08	2.1E-06
path:hsa04727	GABAergic synapse	83	9	5.3E-07	1.4E-05
path:hsa04024	cAMP signaling pathway	192	12	1.1E-06	2.7E-05
path:hsa05224	Breast cancer	143	11	1.9E-06	3.7E-05
path:hsa04916	Melanogenesis	101	9	1.9E-06	3.7E-05
path:hsa04060	Cytokine-cytokine receptor interaction	233	10	1.9E-06	3.7E-05
path:hsa05033	Nicotine addiction	36	6	3.1E-06	5.6E-05
path:hsa05217	Basal cell carcinoma	55	7	6.2E-06	0.00011
path:hsa04726	Serotonergic synapse	107	8	7.9E-06	0.00013
path:hsa05200	Pathways in cancer	386	16	1.1E-05	0.00016

Demethylated in $MB_{Grp4\text{-}HR}$ vs $MB_{Grp4\text{-}LR}$

Pathway ID	Pathway	Total n	no. hypo-methylated	P	AdjP
path:hsa04151	PI3K-Akt signaling pathway	314	21	4.5E-11	1.4E-08
path:hsa01100	Metabolic pathways	1178	30	8.3E-09	1.3E-06
path:hsa05200	Pathways in cancer	386	20	3.3E-08	3.4E-06
path:hsa04390	Hippo signaling pathway	150	13	8.7E-08	5.6E-06
path:hsa04261	Adrenergic signaling in cardiomyocytes	144	12	9.1E-08	5.6E-06
path:hsa04152	AMPK signaling pathway	120	11	3.1E-07	1.6E-05
path:hsa04020	Calcium signaling pathway	171	12	7.8E-07	3.5E-05
path:hsa05206	MicroRNAs in cancer	279	13	1.0E-06	3.9E-05
path:hsa04974	Protein digestion and absorption	78	8	2.1E-06	7.3E-05
path:hsa04360	Axon guidance	170	12	6.9E-06	0.0002
path:hsa05414	Dilated cardiomyopathy	86	8	7.5E-06	0.0002
path:hsa04015	Rap1 signaling pathway	208	12	7.9E-06	0.0002
path:hsa04918	Thyroid hormone synthesis	72	7	1.5E-05	0.0004
path:hsa04340	Hedgehog signaling pathway	46	6	2.2E-05	0.0005
path:hsa05202	Transcriptional misregulation in cancer	165	10	3.2E-05	0.0006
path:hsa04922	Glucagon signaling pathway	96	7	3.2E-05	0.0006
path:hsa04062	Chemokine signaling pathway	178	9	4.0E-05	0.0007
path:hsa04512	ECM-receptor interaction	80	7	5.2E-05	0.0009
path:hsa04310	Wnt signaling pathway	138	9	5.4E-05	0.0009
path:hsa05162	Measles	115	7	6.3E-05	0.0010

Demethylated in $MB_{Grp3\text{-}HR}$ vs $MB_{Grp3\text{-}LR}$

Pathway ID	Pathway	Total n	no. hypo-methylated	P	AdjP
path:hsa01100	Metabolic pathways	1178	89	5.7E-32	1.8E-29
path:hsa04151	PI3K-Akt signaling pathway	314	46	1.5E-24	2.4E-22
path:hsa05200	Pathways in cancer	386	49	1.5E-22	1.6E-20
path:hsa04810	Regulation of actin cytoskeleton	202	34	4.6E-20	3.5E-18
path:hsa04510	Focal adhesion	193	30	3.4E-16	2.1E-14
path:hsa04080	Neuroactive ligand-receptor interaction	255	29	2.7E-15	1.4E-13
path:hsa04024	cAMP signaling pathway	192	26	1.0E-13	4.5E-12
path:hsa04014	Ras signaling pathway	219	27	7.3E-13	2.8E-11
path:hsa04015	Rap1 signaling pathway	208	26	2.8E-12	9.4E-11
path:hsa04010	MAPK signaling pathway	244	28	3.0E-12	9.4E-11
path:hsa04020	Calcium signaling pathway	171	23	7.6E-12	2.1E-10
path:hsa04360	Axon guidance	170	24	2.3E-11	6.1E-10
path:hsa04060	Cytokine-cytokine receptor interaction	233	21	5.7E-11	1.2E-09
path:hsa05146	Amoebiasis	93	16	6.0E-11	1.2E-09
path:hsa05205	Proteoglycans in cancer	200	24	6.1E-11	1.2E-09
path:hsa00230	Purine metabolism	164	20	6.3E-11	1.2E-09
path:hsa05202	Transcriptional misregulation in cancer	165	21	3.0E-10	5.5E-09
path:hsa04062	Chemokine signaling pathway	178	20	3.8E-10	6.6E-09
path:hsa04514	Cell adhesion molecules (CAMs)	133	18	5.0E-10	7.8E-09
path:hsa04512	ECM-receptor interaction	80	15	5.0E-10	7.8E-09

Validation Cohort (Hovestadt *et al*.) (Total n = 276)						
	Pathway ID	Pathway	Total n	no. hypo-methylated	P	AdjP
Demethylated in MB_{WNT} vs normal cerebella	path:hsa04151	PI3K-Akt signaling pathway	314	21	4.5E-11	1.4E-08
	path:hsa01100	Metabolic pathways	1178	30	8.3E-09	1.3E-06
	path:hsa05200	Pathways in cancer	386	20	3.3E-08	3.4E-06
	path:hsa04390	Hippo signaling pathway	150	13	8.7E-08	5.6E-06
	path:hsa04261	Adrenergic signaling in cardiomyocytes	144	12	9.1E-08	5.6E-06
	path:hsa04152	AMPK signaling pathway	120	11	3.1E-07	1.6E-05
	path:hsa04020	Calcium signaling pathway	171	12	7.8E-07	3.5E-05
	path:hsa05206	MicroRNAs in cancer	279	13	1.0E-06	3.9E-05
	path:hsa04974	Protein digestion and absorption	78	8	2.1E-06	7.3E-05
	path:hsa04360	Axon guidance	170	12	6.9E-06	0.0002
	path:hsa05414	Dilated cardiomyopathy	86	8	7.5E-06	0.0002
	path:hsa04015	Rap1 signaling pathway	208	12	7.9E-06	0.0002
	path:hsa04918	Thyroid hormone synthesis	72	7	1.5E-05	0.0004
	path:hsa04340	Hedgehog signaling pathway	46	6	2.2E-05	0.0005
	path:hsa05202	Transcriptional misregulation in cancer	165	10	3.2E-05	0.0006
	path:hsa04922	Glucagon signaling pathway	96	7	3.2E-05	0.0006
	path:hsa04062	Chemokine signaling pathway	178	9	4.0E-05	0.0007
	path:hsa04512	ECM-receptor interaction	80	7	5.2E-05	0.0009
	path:hsa04310	Wnt signaling pathway	138	9	5.4E-05	0.0009
	path:hsa05162	Measles	115	7	6.3E-05	0.0010

	Pathway ID	Pathway	Total n	no. hypo-methylated	P	AdjP
Demethylated in $MB_{SHH\text{-}Child}$ vs $MB_{SHH\text{-}Infant}$	path:hsa04740	Olfactory transduction	357	16	0	0
	path:hsa05332	Graft-versus-host disease	36	3	0.00007	0.0111
	path:hsa04612	Antigen processing and presentation	61	3	0.00024	0.0247
	path:hsa04145	Phagosome	137	3	0.00202	0.1560
	path:hsa05152	Tuberculosis	156	3	0.00252	0.1560
	path:hsa05202	Transcriptional misregulation in cancer	165	3	0.00339	0.1750
	path:hsa05150	Staphylococcus aureus infection	47	2	0.00417	0.1802
	path:hsa05200	Pathways in cancer	386	4	0.00470	0.1802
	path:hsa05140	Leishmaniasis	63	2	0.00523	0.1802
	path:hsa04610	Complement and coagulation cascades	74	2	0.00828	0.2567
	path:hsa05166	HTLV-I infection	250	3	0.00927	0.2611
	path:hsa04974	Protein digestion and absorption	78	2	0.01087	0.2808
	path:hsa05322	Systemic lupus erythematosus	113	2	0.01612	0.3845
	path:hsa04650	Natural killer cell mediated cytotoxicity	106	2	0.01835	0.4062
	path:hsa04141	Protein processing in endoplasmic reticulum	159	2	0.02283	0.4717
	path:hsa04261	Adrenergic signaling in cardiomyocytes	144	2	0.02772	0.5276
	path:hsa05168	Herpes simplex infection	162	2	0.02893	0.5276
	path:hsa00515	Mannose type O-glycan biosynthesis	22	1	0.03388	0.5836
	path:hsa05203	Viral carcinogenesis	191	2	0.04487	0.7321
	path:hsa00051	Fructose and mannose metabolism	32	1	0.05223	0.8038

	Pathway ID	Pathway	Total n	no. hypo-methylated	P	AdjP
Demethylated in $MB_{SHH\text{-}Infant}$ vs $MB_{SHH\text{-}Child}$	path:hsa04080	Neuroactive ligand-receptor interaction	255	27	5.4E-21	1.7E-18
	path:hsa05322	Systemic lupus erythematosus	113	13	2.4E-12	3.7E-10
	path:hsa05034	Alcoholism	164	16	4.8E-12	4.9E-10
	path:hsa04550	Signaling pathways regulating pluripotency of stem cells	138	16	2.7E-11	1.7E-09
	path:hsa04724	Glutamatergic synapse	113	15	2.7E-11	1.7E-09
	path:hsa05202	Transcriptional misregulation in cancer	165	17	3.7E-11	1.9E-09
	path:hsa04950	Maturity onset diabetes of the young	26	8	3.3E-10	1.5E-08
	path:hsa04020	Calcium signaling pathway	171	16	4.7E-10	1.8E-08
	path:hsa05205	Proteoglycans in cancer	200	17	1.1E-09	3.6E-08
	path:hsa04723	Retrograde endocannabinoid signaling	96	12	3.1E-09	9.6E-08
	path:hsa04060	Cytokine-cytokine receptor interaction	233	13	6.3E-09	1.8E-07
	path:hsa05033	Nicotine addiction	36	8	1.4E-08	3.7E-07
	path:hsa04390	Hippo signaling pathway	150	14	2.4E-08	5.6E-07
	path:hsa04916	Melanogenesis	101	11	4.8E-08	1.1E-06
	path:hsa04024	cAMP signaling pathway	192	14	7.2E-08	1.5E-06
	path:hsa05200	Pathways in cancer	386	20	1.2E-07	2.3E-06
	path:hsa01100	Metabolic pathways	1178	29	9.2E-07	1.7E-05
	path:hsa05217	Basal cell carcinoma	55	8	1.0E-06	1.8E-05
	path:hsa05032	Morphine addiction	88	9	4.4E-06	7.2E-05
	path:hsa05224	Breast cancer	143	11	5.3E-06	8.2E-05

Demethylated in $MB_{Grp4\text{-}HR}$ vs $MB_{Grp4\text{-}LR}$

Pathway ID	Pathway	Total n	no. hypo-methylated	P	AdjP
path:hsa05202	Transcriptional misregulation in cancer	165	12	4.3E-08	6.50E-06
path:hsa04151	PI3K-Akt signaling pathway	314	15	8.1E-08	6.50E-06
path:hsa04310	Wnt signaling pathway	138	11	8.2E-08	6.50E-06
path:hsa05200	Pathways in cancer	386	17	8.4E-08	6.50E-06
path:hsa04916	Melanogenesis	101	9	2.5E-07	1.58E-05
path:hsa04360	Axon guidance	170	12	4.4E-07	2.24E-05
path:hsa04015	Rap1 signaling pathway	208	12	5.1E-07	2.24E-05
path:hsa05034	Alcoholism	164	9	1.4E-06	5.25E-05
path:hsa04390	Hippo signaling pathway	150	10	2.8E-06	9.72E-05
path:hsa04014	Ras signaling pathway	219	11	3.7E-06	0.0001
path:hsa01100	Metabolic pathways	1178	21	6.4E-06	0.0002
path:hsa04724	Glutamatergic synapse	113	8	1.6E-05	0.0004
path:hsa05206	MicroRNAs in cancer	279	10	1.7E-05	0.0004
path:hsa05217	Basal cell carcinoma	55	6	2.1E-05	0.0005
path:hsa04918	Thyroid hormone synthesis	72	6	3.4E-05	0.0007
path:hsa04550	Signaling pathways regulating pluripotency of stem cells	138	8	4.2E-05	0.0008
path:hsa04972	Pancreatic secretion	89	6	4.4E-05	0.0008
path:hsa03320	PPAR signaling pathway	69	5	4.8E-05	0.0008
path:hsa04713	Circadian entrainment	95	7	4.8E-05	0.0008
path:hsa04150	mTOR signaling pathway	150	8	5.1E-05	0.0008

Demethylated in $MB_{Grp3\text{-}HR}$ vs $MB_{Grp3\text{-}LR}$

Pathway ID	Pathway	Total n	no. hypo-methylated	P	AdjP
path:hsa01100	Metabolic pathways	1178	22	1.2E-07	2.6E-05
path:hsa04151	PI3K-Akt signaling pathway	314	13	1.7E-07	2.6E-05
path:hsa04713	Circadian entrainment	95	8	5.9E-07	6.1E-05
path:hsa04014	Ras signaling pathway	219	10	2.5E-06	0.0002
path:hsa04024	cAMP signaling pathway	192	9	4.8E-06	0.0003
path:hsa04810	Regulation of actin cytoskeleton	202	9	1.0E-05	0.0005
path:hsa05202	Transcriptional misregulation in cancer	165	8	2.3E-05	0.0010
path:hsa05321	Inflammatory bowel disease (IBD)	61	5	2.5E-05	0.0010
path:hsa04010	MAPK signaling pathway	244	9	5.5E-05	0.0019
path:hsa00450	Selenocompound metabolism	16	3	6.1E-05	0.0019
path:hsa04360	Axon guidance	170	8	7.8E-05	0.0022
path:hsa04611	Platelet activation	121	6	0.0001	0.0027
path:hsa04015	Rap1 signaling pathway	208	8	0.0001	0.0027
path:hsa04020	Calcium signaling pathway	171	7	0.0002	0.0039
path:hsa04610	Complement and coagulation cascades	74	4	0.0002	0.0039
path:hsa05152	Tuberculosis	156	6	0.0002	0.0047
path:hsa04530	Tight junction	133	6	0.0003	0.0047
path:hsa05200	Pathways in cancer	386	10	0.0004	0.0064
path:hsa04310	Wnt signaling pathway	138	6	0.0005	0.0079
path:hsa05205	Proteoglycans in cancer	200	7	0.0006	0.0088

Supplementary Table 2. Pathway enrichment analyses. For each non-MB_{WNT} subgroup, hypomethylated probes with beta change < -0·3 were identified in comparison with their established consensus subgroup counterpart and tested for pathway enrichment using the R package missMethyl. For MB_{WNT}, comparisons were made against normal cerebella. The top 20 pathways are reported for analyses carried out on the discovery and validation cohorts. For $MB_{Grp3\text{-}LR}$ and $MB_{Grp4\text{-}LR}$, no significant pathway enrichments were reported in either cohort, and are not shown here.
